# Baseline low-level viremia in HBV-related hepatocellular carcinoma: prognostic significance, risk stratification, and clinical implications

**DOI:** 10.3389/fimmu.2026.1900644

**Published:** 2026-09-22

**Authors:** Heng Ma, Lihua Yu, Xiaoli Liu, Huiwen Yan, Yuqing Xie, Zimeng Shang, Guangyuan Huo, Wanxin Shi, Zhe Zhang, Zhiyun Yang

**Affiliations:** 1 Center of Integrative Medicine, Beijing Ditan Hospital, Capital Medical University, Beijing, China; 2 Laboratory for Clinical Medicine, Capital Medical University, Beijing, China

**Keywords:** hepatitis B virus, hepatocellular carcinoma, low-level viremia, machine learning, overall survival

## Abstract

**Introduction:**

The prognostic relevance of baseline low-level viremia (LLV) after hepatitis B virus-related hepatocellular carcinoma (HBV-HCC) diagnosis remains uncertain. We evaluated the association between baseline LLV and overall survival (OS), explored related biological features, and developed survival prediction models for patients with baseline LLV.

**Methods:**

This retrospective study included 921 patients with HBV-HCC: 611 with baseline LLV and 310 with HBV DNA <20 IU/mL. Propensity score matching (PSM), inverse probability of treatment weighting (IPTW), and Cox regression were used to assess the association between baseline HBV DNA status and OS. Peripheral T cell subsets and selected markers of hepatic functional reserve, hepatocellular injury, and systemic inflammation were explored. Patients with baseline LLV were randomly divided into training and reserved internal validation sets. Model 1 used the full candidate variable set, whereas Model 2 used non-imaging variables. Predictors were selected using LASSO Cox regression and Boruta, and five survival algorithms were compared within the training set.

**Results:**

Baseline LLV was associated with poorer OS. After PSM, HBV DNA <20 IU/mL was associated with lower adjusted mortality than LLV (hazard ratio, 0.688; 95% confidence interval, 0.543–0.872; P = 0.002), with similar findings after IPTW. Patients with LLV had a lower peripheral CD4/CD8 ratio, and exploratory analyses identified significant indirect associations through albumin, aspartate aminotransferase, and systemic immune-inflammation response index. Model 1 retained tumor size, tumor multiplicity, portal vein tumor thrombus, cholinesterase, alkaline phosphatase, and alpha-fetoprotein; Model 2 retained gamma-glutamyl transferase, cholinesterase, alkaline phosphatase, and alpha-fetoprotein. Random survival forest was selected based on overall training performance. In the reserved validation set, the 1-, 3-, and 5-year time-dependent AUCs were 0.824, 0.777, and 0.765 for Model 1 and 0.769, 0.742, and 0.723 for Model 2, respectively. Both models showed good calibration and positive net benefit.

**Discussion:**

Baseline LLV remained prognostically relevant after HCC diagnosis and was accompanied by exploratory differences in systemic inflammation and the relative balance of peripheral T cell subsets. These findings support closer virological monitoring and individualized risk assessment.

## Introduction

Hepatocellular carcinoma (HCC) remains a leading cause of cancer-related mortality worldwide, and chronic hepatitis B virus (HBV) infection is a major etiological driver, particularly in regions with a high HBV burden (1, 2). The widespread use of nucleos(t)ide analogs has substantially suppressed HBV replication and reduced the risk of cirrhosis and HCC (3, 4). However, antiviral therapy does not eliminate the risk of liver disease progression or HCC development (5, 6). During nucleos(t)ide analog therapy, patients may achieve maintained virological response, commonly characterized by persistently undetectable HBV DNA or HBV DNA <20 IU/mL, whereas others continue to have persistent or intermittent low-level viremia (LLV), usually defined as HBV DNA levels between 20 and 2000 IU/mL (7, 8). Although LLV has traditionally been regarded as a state of relatively controlled viral replication, increasing evidence suggests that it may not represent a benign virological condition.

In chronic HBV infection, LLV has been associated with adverse long-term outcomes. Compared with patients with maintained virological response or undetectable HBV DNA, those with LLV or low but detectable HBV DNA remain at increased risk of HCC development (9–11). Emerging evidence further suggests that low-level HBV replication may retain clinical relevance in patients with established HCC. Among patients with HBV-related HCC (HBV-HCC) receiving locoregional therapy, immune checkpoint inhibitors, or systemic therapy, LLV has been associated with unfavorable recurrence and progression outcomes and poorer overall survival (OS) compared with maintained virological response or HBV DNA <20 IU/mL (12–15). These findings suggest that residual low-level viral replication may remain prognostically relevant after HCC diagnosis, raising the question of whether baseline LLV may identify a subgroup of patients with HBV-HCC who remain at increased residual risk.

Although longitudinal HBV DNA trajectories may provide additional prognostic information during follow-up, baseline HBV DNA is a routinely available measure at the time of initial HCC evaluation. This early virological marker may help identify residual viral activity before subsequent treatment response and follow-up HBV DNA data become available. However, whether baseline LLV is associated with worse survival than baseline HBV DNA <20 IU/mL in a broader HBV-HCC population remains insufficiently clarified. In particular, its prognostic value requires further evaluation across different initial HCC treatment strategies.

The biological mechanisms linking LLV to outcomes in HBV-HCC remain incompletely understood. Persistent viral antigen exposure may contribute to impaired HBV-specific T cell responses and immune phenotypes associated with T cell exhaustion, although these alterations appear to be heterogeneous (7, 16). Emerging evidence also suggests that residual viral activity may be linked to host immune dysregulation and response to immunotherapy (13). Nevertheless, the immune characteristics associated with baseline LLV in patients with HBV-HCC remain poorly defined.

If baseline LLV defines a clinically relevant residual-risk population, prognosis within this subgroup is also unlikely to be homogeneous. Current prognostic assessment in HCC relies largely on conventional staging systems and clinicopathological factors, including Barcelona Clinic Liver Cancer (BCLC) stage, tumor–node–metastasis (TNM) stage, tumor burden, vascular invasion, liver function, and alpha-fetoprotein (AFP) level (17–19). Although these factors are widely used, they were developed for the broader HCC population and may not fully capture prognostic heterogeneity among patients with baseline LLV. Dedicated prediction models for this subgroup may therefore refine individualized survival estimation and follow-up planning.

To address these gaps, we evaluated the association between baseline LLV and OS in a broad cohort of patients with HBV-HCC. We also conducted an exploratory assessment of peripheral blood immune cell characteristics and immune inflammation associated with baseline HBV DNA status. Finally, we developed and internally validated survival prediction models using routinely available clinical data to refine individualized risk assessment among patients with baseline LLV.

## Methods

### Study design and participants

This retrospective study included patients diagnosed with HBV-HCC at Beijing Ditan Hospital between January 2007 and September 2017. Patients were categorized into two groups according to baseline HBV DNA level: the baseline HBV DNA <20 IU/mL group and the baseline LLV group, defined as HBV DNA 20–2000 IU/mL. Patients were excluded if they had HIV (human immunodeficiency virus) infection or other hepatotropic virus co-infection, other primary malignancies, baseline HBV DNA >2000 IU/mL, or were lost to follow-up (**Figure 1**). The study was conducted in accordance with the Declaration of Helsinki and was approved by the institutional ethics committee.

**Figure 1:**
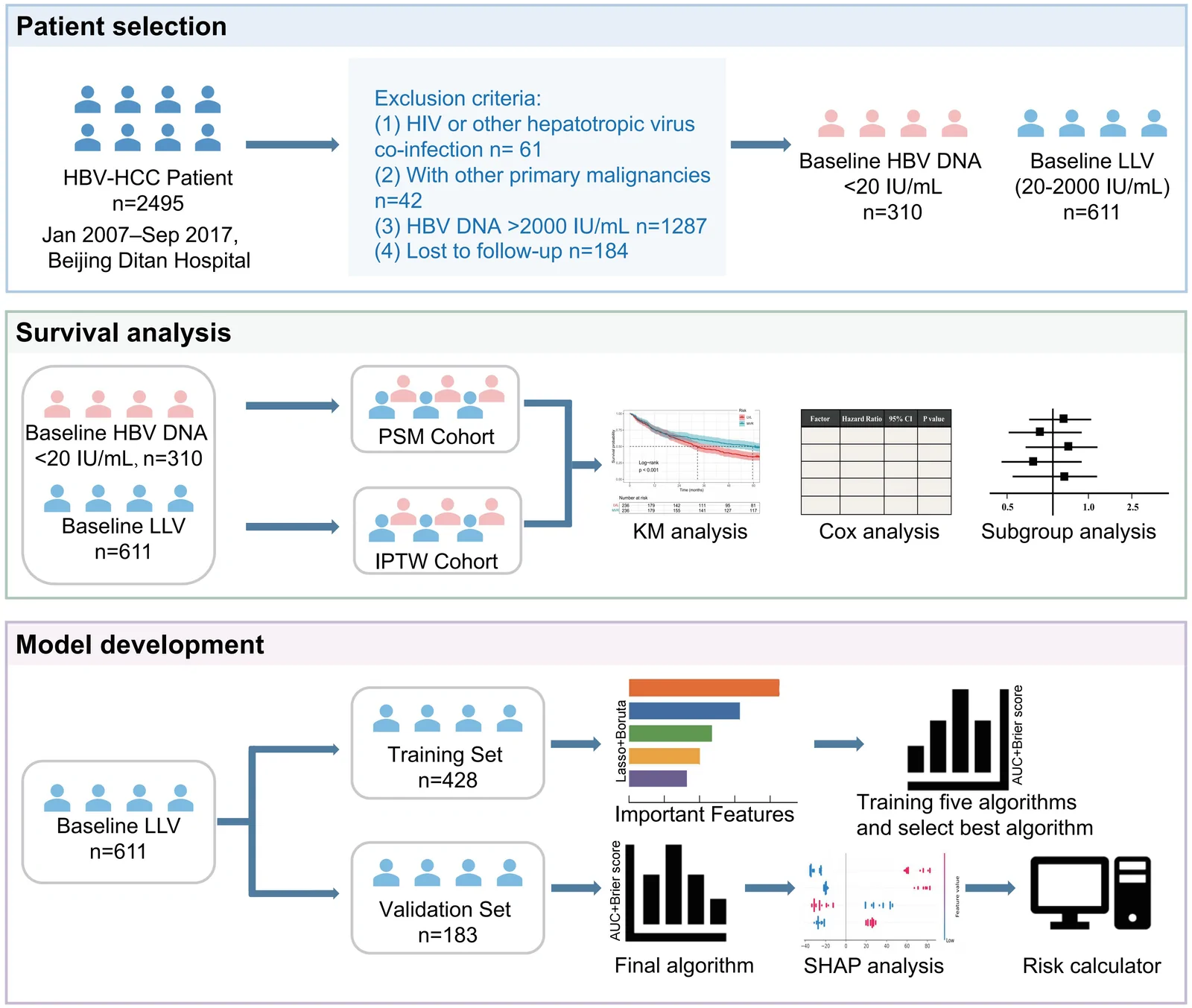
Study flowchart of patient selection, survival analyses, and prediction model development.

### Outcome definition and follow-up

Overall survival (OS) was defined as the interval from the date of initial HCC diagnosis to death from any cause. Vital status and dates of death were ascertained from electronic medical records, outpatient follow-up records, and telephone follow-up with patients or their family members. The administrative end of follow-up was September 2022. Median follow-up duration was estimated using the reverse Kaplan–Meier method.

### Baseline data and missing data handling

Clinical and laboratory variables were obtained from the evaluation associated with the baseline HBV DNA measurement. Study variables were selected according to clinical relevance, availability in routine practice, and previously reported prognostic value in HBV-HCC. Their definitions, classifications, cutoff values, and roles in the analyses are provided in **Supplementary Table S1**.

Variables with less than 15% missingness were eligible for the primary analyses, whereas variables with greater missingness were considered only in exploratory analyses. The distribution of missing data is shown in **Supplementary Figure S1**. For analyses evaluating the association between baseline HBV DNA status and OS, missing baseline covariate values were imputed using a random forest-based method in the full cohort of 921 patients before propensity score and survival analyses. For prediction modeling, missing data were handled only after patients had been divided into the training and validation sets.

### Survival analysis and propensity score adjustment

To reduce baseline imbalance between the baseline HBV DNA <20 IU/mL group and the baseline LLV group, propensity score matching (PSM) and inverse probability of treatment weighting (IPTW) were performed. Propensity scores were estimated using a logistic regression model that included prespecified baseline covariates selected for propensity score adjustment, as detailed in **Supplementary Table S1**. For PSM, patients were matched at a 1:1 ratio using nearest-neighbor matching without replacement and a caliper width equal to 0.1 times the standard deviation of the estimated propensity score distance. Propensity score overlap and covariate balance were evaluated before and after adjustment. An absolute standardized mean difference <0.10 was considered indicative of acceptable balance.

Kaplan–Meier curves were generated to compare OS between groups in the unmatched and PSM cohorts, and differences were evaluated using the log-rank test. Cox proportional hazards models were fitted in the unmatched, propensity score-matched, and IPTW-weighted cohorts. Cluster-robust standard errors based on matched-pair membership were used for the PSM analyses, and robust sandwich variance estimates were used for the IPTW analyses. The multivariable models adjusted for prespecified clinical, virological, liver-related, and tumor-related factors.

Multicollinearity and the proportional hazards assumption were evaluated as model diagnostics. Initial HCC treatment was defined as the first HCC-directed treatment modality recorded during the initial treatment course. All initial HCC treatments occurred after baseline HBV DNA measurement. Because treatment modality was therefore not a strict pre-exposure covariate and a potential mediating role could not be excluded, propensity score and multivariable analyses were repeated after excluding initial HCC treatment as a sensitivity analysis. Subgroup analyses were performed in the matched cohort, and interaction terms were used to examine heterogeneity across clinically relevant strata.

### Prediction model development and evaluation

Prediction models were developed among patients with baseline LLV. Before any data processing for model development, patients were randomly divided in a 7:3 ratio into a training set and a held-out internal validation set. All decisions regarding data processing, feature selection, hyperparameter tuning, and algorithm selection were based only on the training set.

The random forest imputation procedure was fitted using the training data and then applied to the validation data without refitting. The cutoff for baseline HBV DNA within the LLV cohort was determined using X-tile in the training set and then applied unchanged to the validation set.

Two prediction models were developed. Model 1 was derived from the full candidate variable set. Model 2 was derived from a non-imaging candidate variable set that excluded imaging-derived tumor characteristics and was designed as a simplified alternative for settings in which detailed imaging-derived tumor variables are incomplete or difficult to standardize. Feature selection for both models was conducted in the training set using Least absolute shrinkage and selection operator (LASSO) Cox regression with 10-fold cross-validation and the Boruta algorithm. Variables retained by both approaches were used for model development.

Using the selected predictors, five survival modeling algorithms were implemented for each model: Cox proportional hazards model, gradient boosting machine (GBM), support vector machine (SVM), random survival forest (RSF), and extreme gradient boosting (XGBoost). Model fitting, hyperparameter tuning, and algorithm comparison were performed only in the training set. The final algorithm was selected based on the overall balance of discrimination and prediction error across the prespecified evaluation time points in the training set. All model-development decisions were completed before the validation set was evaluated. The candidate parameter ranges, selected settings, random seed, and software specifications are provided in **Supplementary Table S2**.

After model selection, the final models were fitted using the full training set and evaluated in the held-out validation set. The validation set did not contribute to data processing, feature selection, hyperparameter tuning, algorithm selection, or any other development decision.

Discrimination and prediction error were evaluated at 12, 36, and 60 months using time-dependent area under the curve (AUC) and Brier scores. Corresponding 95% confidence intervals were estimated using 1,000 bootstrap resamples. Calibration was evaluated using calibration curves, and potential clinical utility was assessed using decision curve analysis. The final models were also compared with established prognostic systems.

Model interpretability was assessed using SHapley Additive exPlanations (SHAP) after the final algorithm had been selected. SHAP values were calculated for the native prediction output of the selected survival model. Web-based risk calculators were subsequently constructed using the Shiny platform to estimate individualized 1-, 3-, and 5-year survival probabilities.

### Exploratory analyses

Exploratory analyses included evaluation of model performance across initial HCC treatment groups, mediation analyses of selected markers reflecting hepatic functional reserve, hepatocellular injury, and systemic inflammation, and analyses of available peripheral blood T cell subsets data. The immune analysis assessed the comparability of patients with and without available peripheral T cell subsets and compared selected peripheral blood T cell subsets parameters according to baseline HBV DNA status.

### General statistical analysis

Continuous variables were summarized as mean ± standard deviation or median with interquartile range, depending on their distribution, and were compared using Student’s t test or the Mann–Whitney U test, as appropriate. Categorical variables were summarized as frequencies and percentages and compared using the chi-square test or Fisher’s exact test. All analyses were performed using R software, version 4.0.5. All statistical tests were two-sided, and P < 0.05 was considered statistically significant.

## Results

### Baseline characteristics and propensity score diagnostics

The study included 921 patients with HBV-HCC who met the eligibility criteria. Of these, 611 were included in the baseline LLV group and 310 in the baseline HBV DNA <20 IU/mL group. Baseline characteristics in the unmatched and PSM cohorts are shown in **Table 1**, and those in the IPTW-weighted cohort are shown in **Supplementary Table S3**.

**Table 1:** Baseline characteristics according to baseline HBV DNA status before and after propensity score matching.

Variable	Unmatched cohort				PSM cohort			
	20-2000 IU/mL	<20 IU/mL	P	SMD	20–2000 IU/mL	<20 IU/mL	P	SMD
N	611	310			236	236		
**Sex = male (%)**	469(76.8)	241(77.7)	0.801	0.023	181(76.7)	183(77.5)	0.913	0.020
**Age >60 (%)**	190(31.1)	113(36.5)	0.119	**0.113**	82(34.7)	79(33.5)	0.846	0.027
**Hypertension = yes (%)**	139(22.7)	77 (24.8)	0.532	0.049	61(25.8)	58(24.6)	0.832	0.029
**Diabetes = yes (%)**	112(18.3)	77 (24.8)	0.026	**0.159**	56(23.7)	57(24.2)	1.000	0.010
**Cirrhosis = yes (%)**	562(92.0)	300(96.8)	0.008	**0.209**	228(96.6)	227(96.2)	1.000	0.023
**BCLC = C-D (%)**	218(35.7)	48 (15.5)	<0.001	**0.476**	43 (18.2)	41 (17.4)	0.904	0.022
**Child-Pugh = B-C (%)**	210(34.4)	85 (27.4)	0.039	**0.151**	68 (28.8)	70 (29.7)	0.919	0.019
**HBsAg >250IU/mL (%)**	453(74.1)	172(55.5)	<0.001	**0.398**	158(66.9)	149(63.1)	0.440	0.080
**HBeAg ≥1IU/mL (%)**	214(35.0)	74 (23.9)	0.001	**0.247**	59 (25.0)	65 (27.5)	0.601	0.058
**Antiviral regimen = First-line drugs (%)**	398(65.1)	208(67.1)	0.604	0.041	158(66.9)	161(68.2)	0.844	0.027
**Initial HCC treatment (%)**			0.364	0.100			0.987	0.015
Systemic Therapy	78 (12.8)	31 (10.0)			21 (8.9)	21 (8.9)		
Minimally Invasive Therapy	484(79.2)	249(80.3)			194(82.2)	195(82.6)		
Surgical Therapy	49 (8.0)	30 (9.7)			21 (8.9)	20 (8.5)		
**Tumor size (%)**			<0.001	**0.614**			0.840	0.054
<3cm	201(32.9)	184(59.4)			125(53.0)	119(50.4)		
3-5cm	176(28.8)	77 (24.8)			66 (28.0)	68 (28.8)		
>5cm	234(38.3)	49 (15.8)			45 (19.1)	49 (20.8)		
**Tumor multiplicity** **= Multiple (%)**	331(54.2)	115(37.1)	<0.001	**0.348**	102(43.2)	98 (41.5)	0.780	0.034
**PVTT = yes (%)**	137(22.4)	35 (11.3)	<0.001	**0.301**	30 (12.7)	29 (12.3)	1.000	0.013
**NLR >2 (%)**	393(64.3)	170(54.8)	0.007	**0.194**	133(56.4)	138(58.5)	0.710	0.043
**HGB <110g/L (%)**	145(23.7)	79 (25.5)	0.614	0.041	56 (23.7)	59 (25.0)	0.830	0.030
**PLT <100 × 10^9/L (%)**	316(51.7)	181(58.4)	0.064	**0.134**	137(58.1)	136(57.6)	1.000	0.009
**Cr >111μmol/L (%)**	16 (2.6)	14 (4.5)	0.181	**0.102**	9 (3.8)	7 (3.0)	0.799	0.047
**ALT >50U/L (%)**	157(25.7)	34 (11.0)	<0.001	**0.388**	36 (15.3)	33 (14.0)	0.794	0.036
**AST >40U/L (%)**	345(56.5)	70 (22.6)	<0.001	**0.739**	74 (31.4)	70 (29.7)	0.764	0.037
**TBIL >18.8umol/L (%)**	298(48.8)	117(37.7)	0.002	**0.224**	99 (41.9)	96 (40.7)	0.852	0.026
**ALB <40g/L (%)**	427(69.9)	185(59.7)	0.002	**0.215**	152(64.4)	147(62.3)	0.702	0.044
**LDH >250U/L (%)**	69 (11.3)	27 (8.7)	0.272	0.086	17 (7.2)	20 (8.5)	0.732	0.047
**GGT >60U/L (%)**	333(54.5)	96 (31.0)	<0.001	**0.490**	80 (33.9)	81 (34.3)	1.000	0.009
**ALP >125U/L (%)**	189(30.9)	54 (17.4)	<0.001	**0.320**	44 (18.6)	47 (19.9)	0.815	0.032
**CHE >4000U/L (%)**	364(59.6)	219(70.6)	0.001	**0.234**	155(65.7)	165(69.9)	0.375	0.091
**INR >1.5 (%)**	42 (6.9)	17 (5.5)	0.502	0.058	13 (5.5)	14 (5.9)	1.000	0.018
**AFP >400ng/mL (%)**	169(27.7)	33 (10.6)	<0.001	**0.443**	35 (14.8)	31(13.1)	0.691	0.049

Values are presented as No. (%) unless otherwise indicated. HBV DNA groups are expressed in IU/mL; 20 -2000 IU/mL represents baseline low-level viremia. Propensity score matching was performed at a 1:1 ratio, and standardized mean differences <0.1 indicate acceptable balance. The bold values in **Table 1** indicate standardized mean differences (SMDs) greater than 0.1. Initial HCC treatment refers to the first HCC-directed treatment modality at baseline.

BCLC, Barcelona Clinic Liver Cancer; HBsAg, hepatitis B surface antigen; HBeAg, hepatitis B e antigen; HCC, hepatocellular carcinoma; PVTT, portal vein tumor thrombus; NLR, neutrophil-tolymphocyte ratio; HGB, hemoglobin; PLT, platelet count; Cr, creatinine; ALT, alanine aminotransferase; AST, aspartate aminotransferase; TBIL, total bilirubin; ALB, albumin; LDH, lactate dehydrogenase; GGT, gamma-glutamyl transferase; ALP, alkaline phosphatase; CHE, cholinesterase; INR, international normalized ratio; AFP, alpha-fetoprotein; PSM, propensity score matching; SMD, standardized mean difference.

Before adjustment, the baseline LLV group had a less favorable clinical profile than the baseline HBV DNA <20 IU/mL group. Patients with baseline LLV more frequently had BCLC stage C–D disease (35.7% vs. 15.5%) and Child–Pugh class B–C (34.4% vs. 27.4%). Markers of HBV activity were also more frequently elevated in the baseline LLV group, including hepatitis B surface antigen (HBsAg) >250 IU/mL (74.1% vs. 55.5%) and hepatitis B e antigen (HBeAg) ≥1 IU/mL (35.0% vs. 23.9%). Tumor burden was greater in this group, as reflected by higher proportions of tumor size >5 cm (38.3% vs. 15.8%), multiple tumors (54.2% vs. 37.1%), portal vein tumor thrombus (PVTT; 22.4% vs. 11.3%), and AFP >400 ng/mL (27.7% vs. 10.6%). The baseline LLV group also had higher frequencies of unfavorable laboratory profiles, including elevated transaminases and cholestatic markers, hypoalbuminemia, and reduced cholinesterase (CHE). In contrast, the baseline HBV DNA <20 IU/mL group had slightly higher proportions of patients aged >60 years (36.5% vs. 31.1%), with diabetes mellitus (24.8% vs. 18.3%), and with cirrhosis (96.8% vs. 92.0%). Several variables, including sex, hypertension, antiviral regimen, hemoglobin (HGB), lactate dehydrogenase (LDH), and international normalized ratio (INR), were balanced before adjustment, with SMDs <0.1.

The propensity score distributions showed substantial overlap between the two groups. The descriptive common-support interval was 0.040–0.872, and 470 of 472 matched patients (99.6%) were within this interval (**Supplementary Figure S2**). After 1:1 PSM, 236 patients remained in each group, and all covariates included in the propensity score model achieved acceptable balance, with absolute SMDs <0.10. After IPTW, most covariates were balanced, although residual imbalance remained for HBsAg, AST, and tumor size, with absolute SMDs >0.10 (**Supplementary Table S3**).

### Association between baseline HBV DNA status and OS

The median follow-up duration, estimated using the reverse Kaplan–Meier method, was 86.2 months (95% CI, 83.5–91.0). During follow-up, 657 deaths occurred among the 921 patients, including 484/611 (79.2%) patients in the baseline LLV group and 173/310 (55.8%) patients in the baseline HBV DNA <20 IU/mL group.

Kaplan–Meier analysis showed significantly worse OS in the baseline LLV group than in the baseline HBV DNA <20 IU/mL group (**Figure 2**). This difference was observed in the unmatched cohort (**Figure 2A**; log-rank P < 0.001) and remained significant after 1:1 PSM (**Figure 2B**; log-rank P < 0.001).

**Figure 2:**
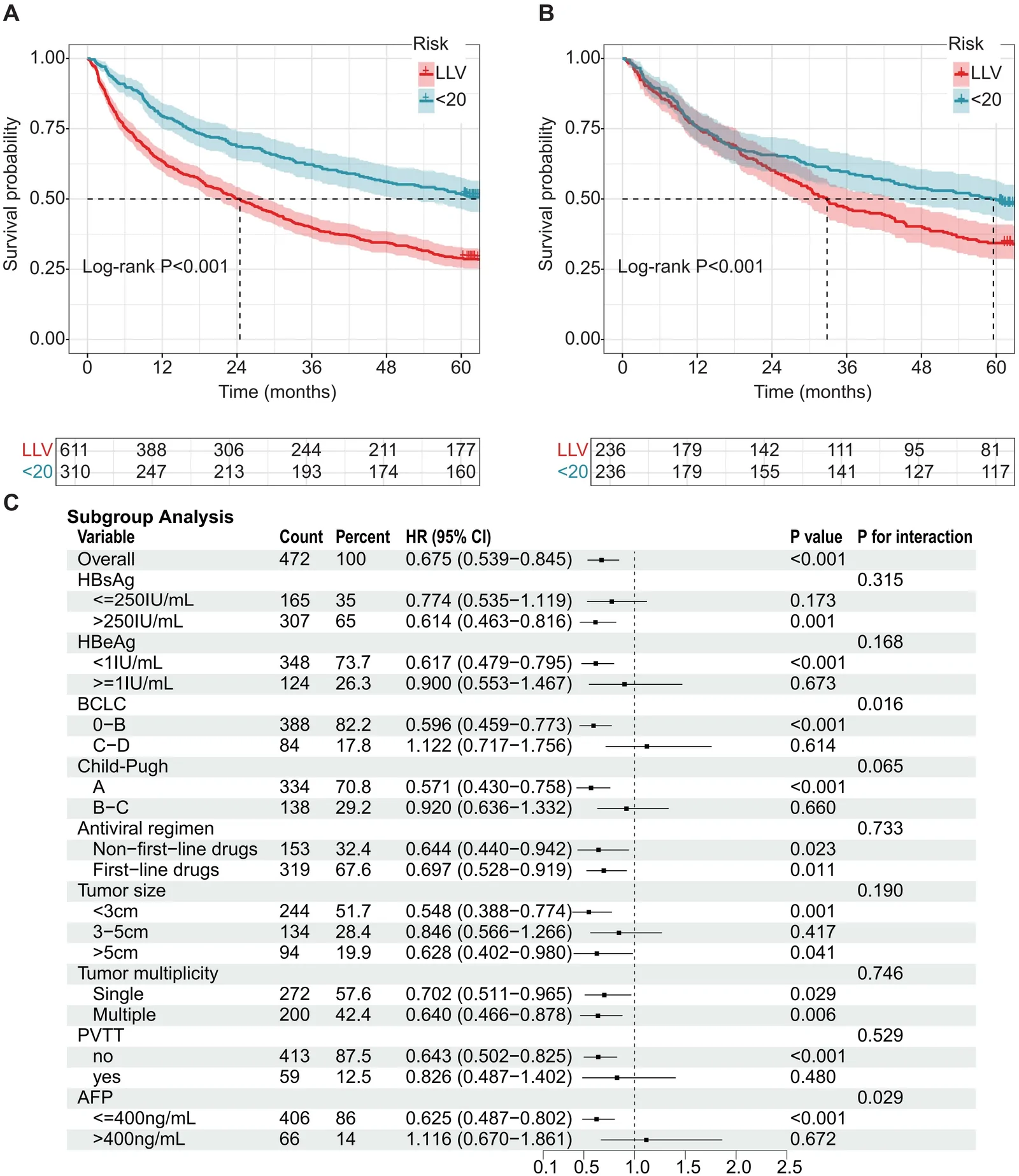
Kaplan–Meier and subgroup analyses of overall survival according to baseline HBV DNA status. Kaplan–Meier curves comparing patients with baseline low-level viremia and those with baseline HBV DNA <20 IU/mL in the unmatched cohort **(A)** and propensity score-matched cohort **(B)**. Subgroup analyses of overall survival in the propensity score-matched cohort are shown in **(C)**. LLV was defined as baseline HBV DNA 20–2000 IU/mL; <20 indicates baseline HBV DNA <20 IU/mL.

Cox proportional hazards models further supported this association across the unmatched, PSM, and IPTW-weighted cohorts (**Table 2**). With the baseline LLV group as the reference, HBV DNA <20 IU/mL was associated with a lower risk of death in the unmatched cohort in both univariable analysis (hazard ratio [HR], 0.546; 95% confidence interval [CI], 0.459–0.650; P < 0.001) and multivariable analysis (HR, 0.751; 95% CI, 0.614–0.918; P = 0.005). Consistent associations were observed after PSM, with HRs of 0.675 (95% CI, 0.539–0.845; P < 0.001) in the univariable model and 0.688 (95% CI, 0.543–0.872; P = 0.002) in the multivariable model. In the IPTW-weighted cohort, the corresponding HRs were 0.672 (95% CI, 0.553–0.816; P < 0.001) and 0.716 (95% CI, 0.583–0.879; P = 0.002), respectively. After additional adjustment for covariates with residual imbalance after weighting, including tumor size, HBsAg, and AST, the association remained significant (HR, 0.722; 95% CI, 0.590–0.883; P = 0.002).

**Table 2:** Association between baseline HBV DNA status and overall survival in the unmatched, propensity score–matched, and IPTW-weighted cohorts.

Statistical model	Hazard ratio	95% CI	P value
Unmatched Cohort			
Univariable Cox	0.546	0.459-0.650	<0.001
Multivariable Cox	0.751	0.614-0.918	0.005
PSM Cohort			
Univariable Cox	0.675	0.539-0.845	<0.001
Multivariable Cox	0.688	0.543-0.872	0.002
IPTW Cohort			
Univariable Cox	0.672	0.553-0.816	<0.001
Multivariable Cox	0.716	0.583-0.879	0.002
Multivariable Cox adjusted for residual imbalance	0.722	0.590-0.883	0.002

Hazard ratios were calculated for patients with baseline HBV DNA <20 IU/mL using patients with baseline HBV DNA 20–2000 IU/mL as the reference group. Multivariable Cox models were adjusted for prespecified baseline covariates. In the IPTW cohort, the additional multivariable Cox model was further adjusted for covariates with residual imbalance after weighting, defined as SMD >0.1.

CI, confidence interval; HBV, hepatitis B virus; IPTW, inverse probability of treatment weighting; PSM, propensity score matching; SMD, standardized mean difference.

No substantial multicollinearity was identified among covariates in the primary multivariable Cox model (variance inflation factor [VIF] range, 1.094–2.694; tolerance range, 0.371–0.914; **Supplementary Table S4**). Baseline HBV DNA status did not show evidence of nonproportional hazards in any primary model (all P ≥ 0.051). After stratification by selected covariates with evidence of nonproportionality, the global proportional hazards tests were no longer statistically significant (P = 0.066–0.720). The association between baseline HBV DNA status and OS remained directionally consistent and statistically significant in these sensitivity analyses, supporting the robustness of the primary findings (**Supplementary Table S5**).

Sensitivity analyses excluding initial HCC treatment from the propensity score and multivariable adjustment sets yielded estimates similar to those of the primary analyses. The adjusted HRs were 0.761 (95% CI, 0.623–0.930; P = 0.007) in the unmatched cohort, 0.703 (95% CI, 0.551–0.898; P = 0.005) in the rematched cohort, and 0.722 (95% CI, 0.586 -0.889; P = 0.001) in the reweighted cohort. Reestimation of the propensity scores produced 241 matched pairs, and all absolute SMDs after PSM and IPTW were ≤0.097 (**Supplementary Table S6**).

Exploratory subgroup analyses in the PSM cohort showed that the association between baseline HBV DNA status and OS was generally consistent across antiviral regimen and tumor multiplicity subgroups (**Figure 2C**). The lower mortality risk associated with HBV DNA <20 IU/mL was observed among patients receiving first-line antiviral drugs (HR, 0.697; 95% CI, 0.528–0.919; P = 0.011) and non-first-line antiviral drugs (HR, 0.644; 95% CI, 0.440–0.942; P = 0.023), as well as among patients with solitary tumors (HR, 0.702; 95% CI, 0.511–0.965; P = 0.029) and multiple tumors (HR, 0.640; 95% CI, 0.466–0.878; P = 0.006).

The magnitude of the association varied across selected clinical strata. The association was more evident among patients with BCLC stage 0–B disease (HR, 0.596; 95% CI, 0.459–0.773; P < 0.001; P for interaction = 0.016) and AFP ≤400 ng/mL (HR, 0.625; 95% CI, 0.487–0.802; P < 0.001; P for interaction = 0.029), whereas no significant association was observed among patients with BCLC stage C–D disease or AFP >400 ng/mL. Overall, these analyses indicated that baseline LLV was consistently associated with worse OS in patients with HBV-HCC.

### Exploratory pathway and peripheral T cell analyses

Exploratory mediation analyses were performed for selected markers reflecting hepatic functional reserve, hepatocellular injury, and systemic inflammation (**Supplementary Figure S3**). Statistically significant natural indirect effects were observed through ALB, AST, and systemic immune-inflammation response index (SIRI). The indirect effect through ALB was HR 1.045 (95% CI, 1.007–1.085; P = 0.019), with an estimated proportion mediated of 13.4%. The corresponding indirect effect through AST was HR 1.073 (95% CI, 1.020–1.130; P = 0.006), with a proportion mediated of 23.1%, whereas that through SIRI was HR 1.032 (95% CI, 1.002–1.062; P = 0.035), with a proportion mediated of 9.6%. In contrast, the indirect effects through CHE, ALT, and NLR were not statistically significant (P = 0.086, 0.482, and 0.266, respectively). These analyses were exploratory and indicate that hepatic functional impairment, hepatocellular injury, and systemic inflammation may statistically account for part of the association between baseline LLV and OS. Of the 921 patients, 531 had available peripheral T cell data and were included in the exploratory immune analysis, including 362 patients with baseline LLV and 169 with HBV DNA <20 IU/mL. The peripheral CD4/CD8 ratio was lower in the LLV group than in the HBV DNA <20 IU/mL group [median (IQR), 1.69 (1.29–2.22) vs. 1.87 (1.36–2.67); P = 0.016; FDR-adjusted P = 0.047]. In contrast, absolute CD4^+^ T cell counts [475.50 (302.75–672.50) vs. 462.00 (303.00–693.00); FDR-adjusted P = 0.684] and absolute CD8^+^ T cell counts [279.00 (162.00–432.25) vs. 256.00 (156.00–365.00); FDR-adjusted P = 0.278] were similar between the two groups (**Supplementary Table S7**).

### Feature selection

Given the adverse prognosis associated with baseline LLV, prediction modeling focused on further risk stratification within the LLV cohort. The 611 patients were randomly divided at a 7:3 ratio into a training set (n=428) and a held-out internal validation set (n=183). Their baseline characteristics are presented in **Supplementary Table S8**.

In the training set, X-tile identified 1600 IU/mL as the cutoff for baseline HBV DNA, and this cutoff was subsequently applied unchanged to the validation set. For Model 1, derived from the full candidate variable set, LASSO regression selected tumor size, tumor multiplicity, PVTT, CHE, alkaline phosphatase (ALP), and AFP (**Figures 3A, B**). Boruta identified tumor size, PVTT, tumor multiplicity, AFP, ALP, gamma-glutamyl transferase (GGT), CHE, initial HCC treatment, LDH, HGB, and total bilirubin (TBIL) as important features (**Figure 3C**). The overlapping variables selected by both methods were retained as final predictors for Model 1: tumor size, tumor multiplicity, PVTT, CHE, ALP, and AFP. Using the same feature selection strategy, Model 2 was derived from the non-imaging candidate variable set. GGT, ALP, AFP, and CHE were retained as final predictors for Model 2 (**Supplementary Figures S4A–C**).

**Figure 3:**
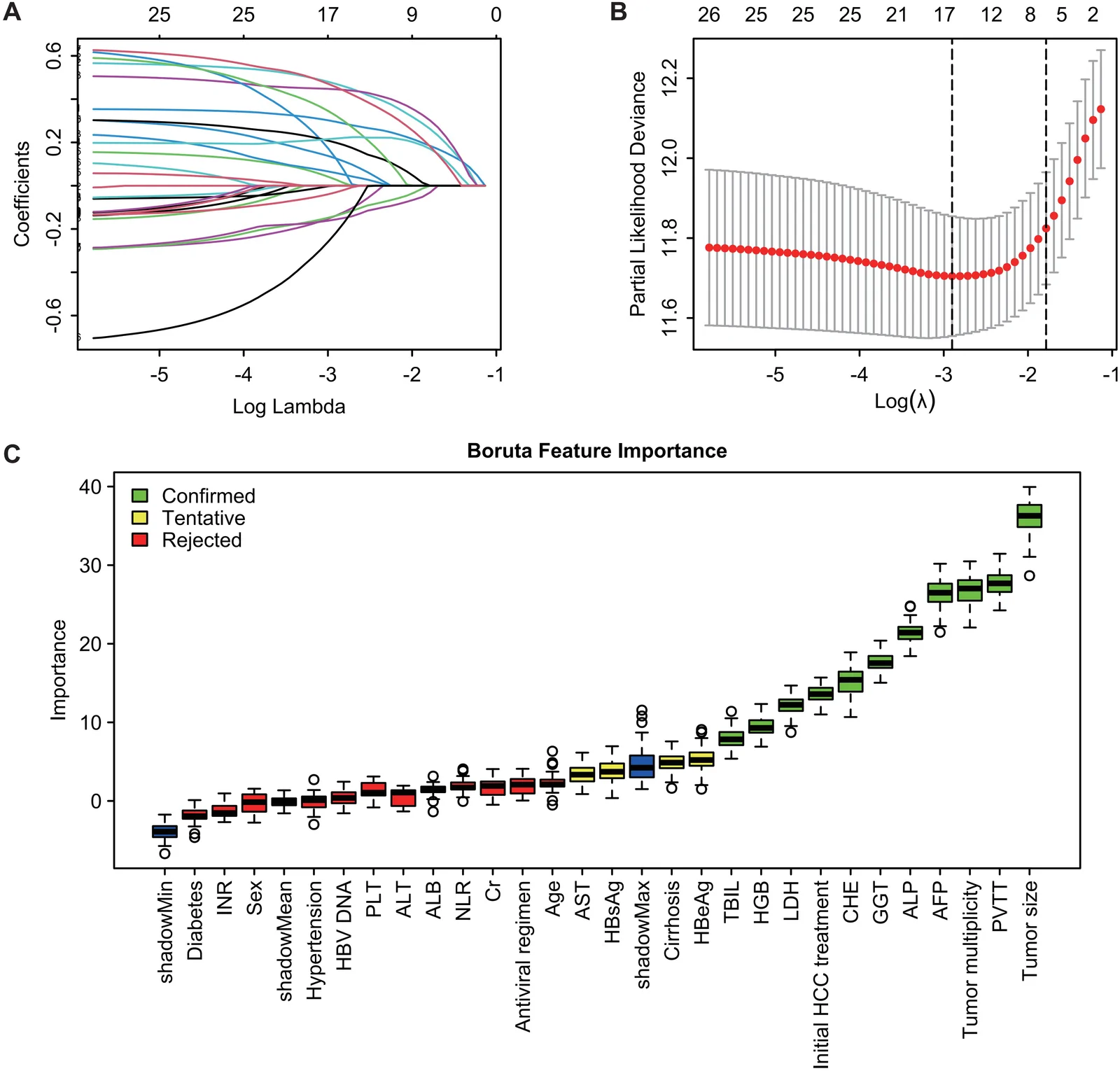
Selection of prognostic features for Model 1 from the full candidate variable set. **(A)** Least absolute shrinkage and selection operator Cox regression coefficient profiles. **(B)** Ten-fold cross-validation for selecting the optimal lambda in the LASSO-Cox model. **(C)** Variable importance ranking generated by the Boruta algorithm.

### Model development and selection

Based on the selected predictors, the five candidate survival algorithms were tuned and compared exclusively within the training set. For Model 1, RSF achieved the highest AUCs at 12 and 36 months (0.842 and 0.825, respectively), remained competitive at 60 months (0.805), and yielded the lowest Brier scores at all three time points (0.157, 0.167, and 0.151). For Model 2, RSF similarly achieved the highest AUCs at 12 and 36 months (0.785 and 0.755), an AUC of 0.729 at 60 months, and the lowest Brier scores at 12, 36, and 60 months (0.174, 0.193, and 0.174). (**Table 3**; **Supplementary Table S9**) According to the prespecified training-set selection rule described in the Methods, RSF was selected as the final algorithm for both models before the validation set was evaluated.

**Table 3:** AUC comparison of survival models in the training and validation sets using 1,000 bootstrap replicates.

Model	Parameters	Training set			Validation set		
		12-month	36-month	60-month	12-month	36-month	60-month
Cox	6	0.826(0.785-0.866)	0.811(0.769-0.852)	0.796(0.752-0.837)	0.815(0.793-0.834)	0.771(0.757-0.783)	0.749(0.735-0.761)
GBM	6	0.836(0.797-0.874)	0.823(0.792-0.862)	0.813(0.774-0.850)	0.813(0.792-0.833)	0.774(0.759-0.788)	0.753(0.733-0.773)
SVM	6	0.793(0.750-0.834)	0.768(0.722-0.813)	0.755(0.702-0.801)	0.789(0.708-0.855)	0.728(0.656-0.796)	0.709(0.628-0.781)
RSF	6	0.842(0.804-0.877)	0.825(0.782-0.862)	0.805(0.761-0.846)	0.824(0.800-0.839)	0.777(0.763-0.790)	0.765(0.750-0.778)
XGB	6	0.830(0.786-0.872)	0.821(0.780-0.863)	0.811(0.765-0.852)	0.807(0.781-0.832)	0.767(0.745-0.787)	0.746(0.717-0.771)
Cox	4	0.773(0.721-0.817)	0.744(0.694-0.792)	0.720(0.665-0.770)	0.764(0.730-0.786)	0.734(0.700-0.754)	0.719(0.690-0.733)
GBM	4	0.781(0.729-0.822)	0.753(0.707-0.800)	0.730(0.676-0.776)	0.762(0.728-0.784)	0.738(0.703-0.757)	0.721(0.691-0.737)
SVM	4	0.776(0.732-0.820)	0.737(0.690-0.785)	0.706(0.649-0.755)	0.789(0.716-0.850)	0.731(0.657-0.801)	0.715(0.634-0.789)
RSF	4	0.785(0.739-0.826)	0.755(0.710-0.802)	0.729(0.675-0.777)	0.769(0.741-0.788)	0.742(0.709-0.757)	0.723(0.694-0.735)
XGB	4	0.768(0.708-0.816)	0.739(0.688-0.788)	0.715(0.657-0.765)	0.757(0.701-0.784)	0.718(0.665-0.744)	0.705(0.660-0.728)

Higher AUC indicates better discriminative performance. Values are mean (95% confidence interval).

AUC, area under the curve; Cox, Cox proportional hazards model; GBM, gradient boosting machine; RSF, random survival forest; SVM, support vector machine; XGB, extreme gradient boosting.

After algorithm selection, the final RSF models were evaluated in the held-out validation set without further tuning or model selection. For Model 1, the time-dependent AUCs at 12, 36, and 60 months were 0.824, 0.777, and 0.765, with corresponding Brier scores of 0.161, 0.190, and 0.179. For Model 2, the corresponding AUCs were 0.769, 0.742, and 0.723, and the Brier scores were 0.179, 0.198, and 0.184 (**Table 3**; **Supplementary Table S9**). These values constituted the prespecified held-out internal validation estimates.

### Calibration, clinical utility, and comparison with conventional prognostic systems

Calibration curves showed generally close agreement between predicted and observed survival probabilities for both models at 12, 36, and 60 months in the training and validation sets (**Figure 4**). Decision curve analysis indicated positive net benefit for both models across a range of threshold probabilities at each time point (**Figure 5**), suggesting potential clinical utility for individualized risk stratification.

**Figure 4:**
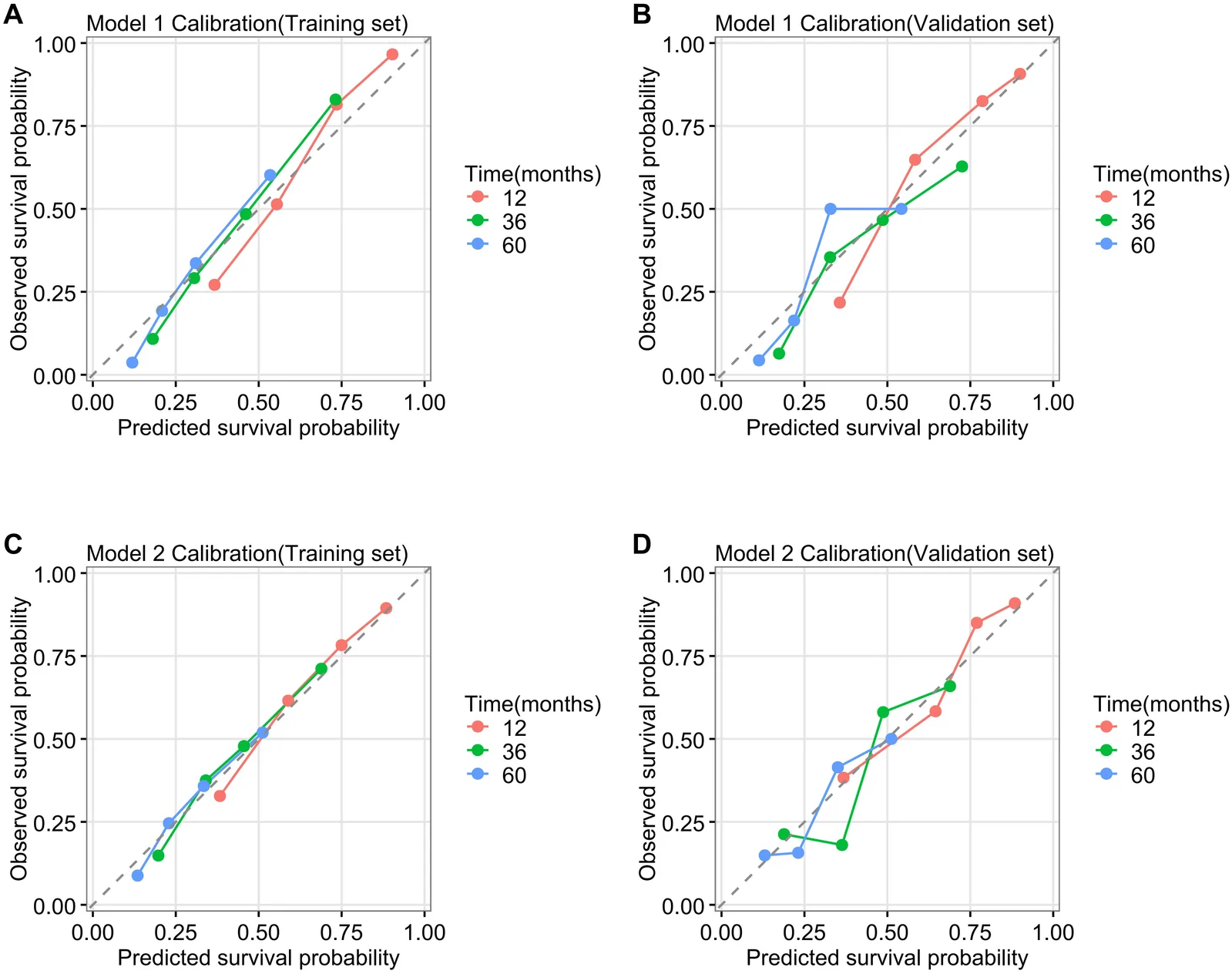
Calibration curves of Model 1 and Model 2 for 12-, 36-, and 60-month overall survival prediction. Calibration curves show the agreement between predicted and observed survival probabilities. **(A)** Model 1 in the training set. **(B)** Model 1 in the validation set. **(C)** Model 2 in the training set. **(D)** Model 2 in the validation set.

**Figure 5:**
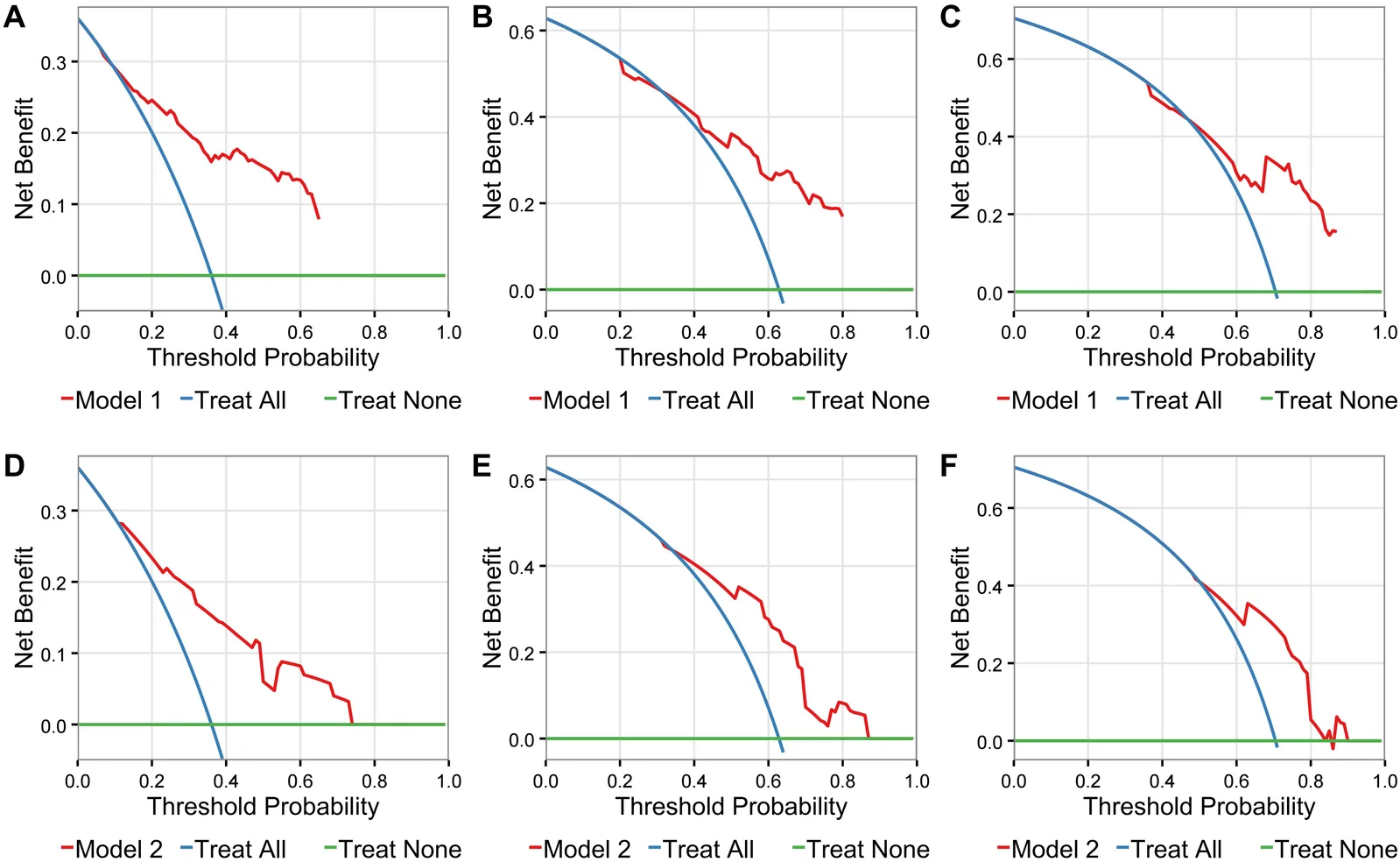
Decision curve analysis of Model 1 and Model 2 for 12-, 36-, and 60-month overall survival prediction in the validation set. Decision curves show the net benefit of Model 1 and Model 2 across threshold probabilities. **(A–C)** Model 1 at 12, 36, and 60 months, respectively. **(D–F)** Model 2 at 12, 36, and 60 months, respectively.

The final RSF models were further compared with BCLC stage, TNM stage, Child–Pugh class, MELD score, and ALBI grade (**Figure 6**; **Supplementary Table S10**). In both the training and validation sets, Model 1 and Model 2 had numerically higher time-dependent AUCs and generally lower Brier scores than the conventional prognostic systems at 12, 36, and 60 months. Model 1 showed the most favorable overall numerical performance.

**Figure 6:**
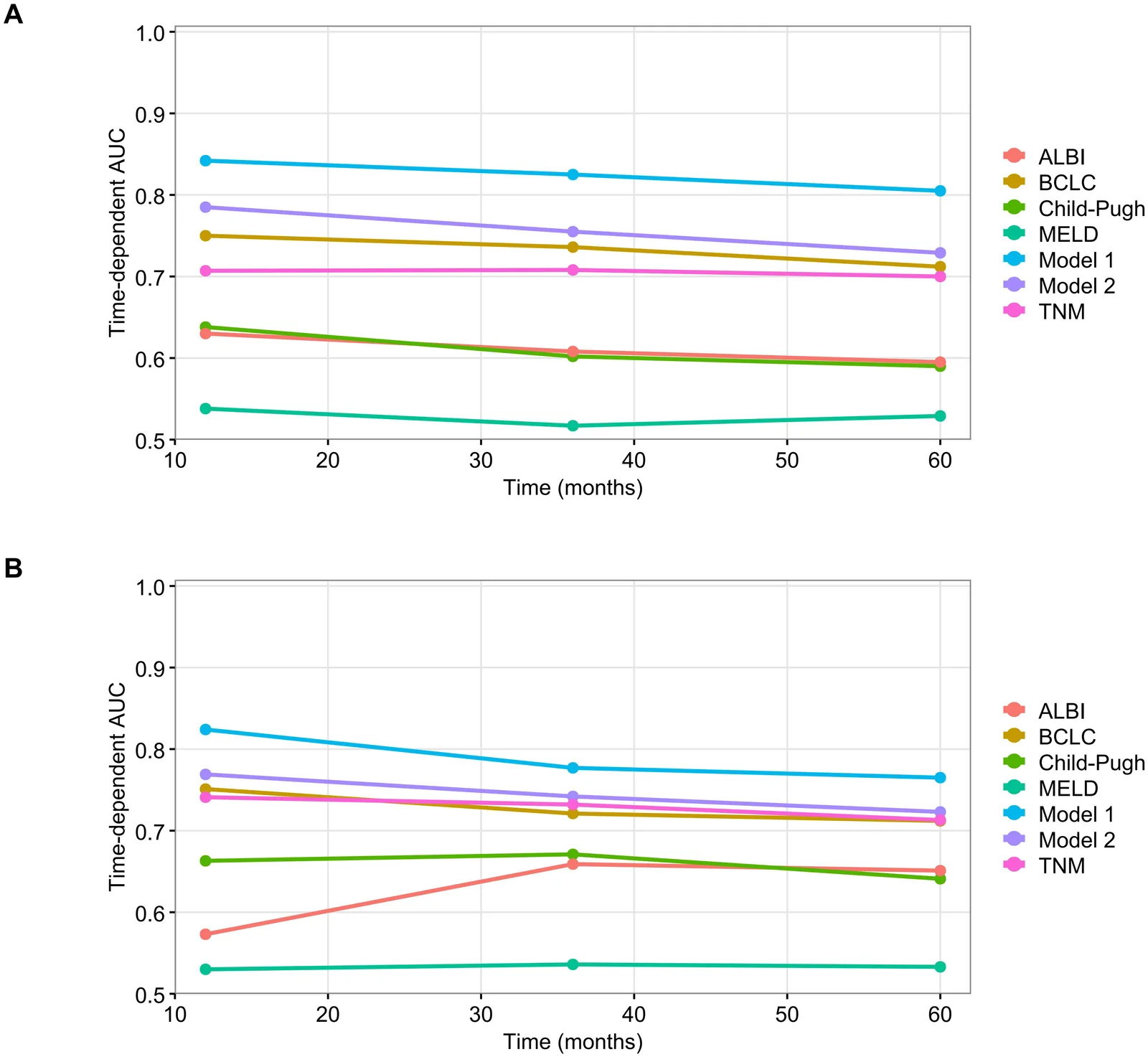
Time-dependent AUC curves comparing Model 1, Model 2, and conventional prognostic systems in the training and validation sets. Time-dependent AUC curves were used to compare the discriminative performance of Model 1, Model 2, and conventional prognostic systems, including BCLC stage, TNM stage, MELD score, ALBI grade, and Child–Pugh class. **(A)** Training set. **(B)** Validation set.

### Exploratory assessment across initial HCC treatment groups

Model performance was explored across surgical therapy (n=49), systemic therapy (n=78), and minimally invasive therapy (n=484) groups (**Supplementary Table S11**). At 12 months, discrimination was highest in the surgical group, with AUCs of 0.871 for Model 1 and 0.813 for Model 2. Performance at 36 and 60 months varied across treatment groups, and Model 1 generally showed more favorable discrimination and prediction error in the minimally invasive group. Given the small surgical and systemic subgroups and the inclusion of both training and validation participants, these analyses were exploratory and did not constitute independent validation.

### Model interpretability and web-based risk calculators

SHAP values quantified feature-specific contributions to the time-independent prognostic risk score returned by the final RSF model. Positive SHAP values indicated higher predicted risk, whereas negative values indicated lower predicted risk. Mean absolute SHAP values reflected the magnitude of feature contributions irrespective of direction.

In Model 1, tumor size showed the highest global importance, with a mean absolute SHAP value of 45.26, followed by tumor multiplicity (30.84), PVTT (29.52), AFP (22.50), ALP (21.83), and CHE (14.46) (**Figure 7A**). In the SHAP distribution plot, tumor size >5 cm, multiple tumors, presence of PVTT, AFP >400 ng/mL, and ALP >125 U/L were predominantly associated with positive SHAP values and higher RSF-predicted risk scores, whereas CHE >4000 U/L was associated with lower predicted risk (**Figure 7B**).

**Figure 7:**
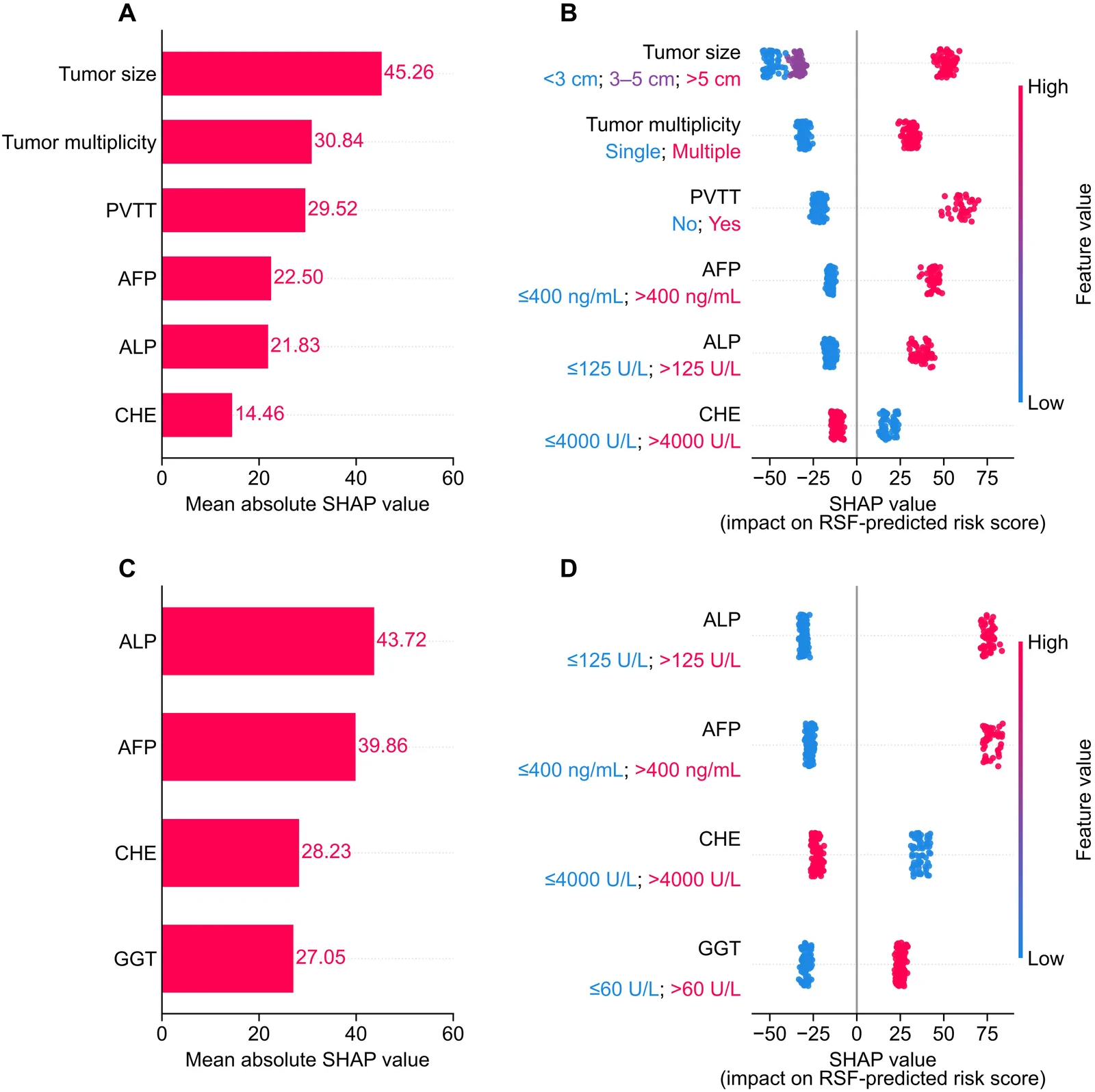
SHAP-based interpretation of the final RSF implementations of Model 1 and Model 2. SHAP values were calculated for the time-independent prognostic risk score returned by the final RSF model. Positive SHAP values indicate contributions toward higher model-predicted risk, whereas negative SHAP values indicate contributions toward lower model-predicted risk. Mean absolute SHAP values represent global feature importance irrespective of direction. **(A)** Bar plot of mean absolute SHAP values for Model 1. **(B)** SHAP summary plot showing the direction and magnitude of feature contributions for Model 1. **(C)** Bar plot of mean absolute SHAP values for Model 2. **(D)** SHAP summary plot showing the direction and magnitude of feature contributions for Model 2.

In Model 2, ALP had the highest mean absolute SHAP value (43.72), followed by AFP (39.86), CHE (28.23), and GGT (27.05) (**Figure 7C**). In the SHAP distribution plot, ALP >125 U/L, AFP >400 ng/mL, and GGT >60 U/L were predominantly associated with positive SHAP values and higher RSF-predicted risk scores, whereas CHE >4000 U/L was associated with negative SHAP values and lower predicted risk (**Figure 7D**).

Based on the final RSF implementations of Model 1 and Model 2, two interactive web-based risk calculators were developed using the Shiny platform. These tools allow real-time estimation of individualized 1-, 3-, and 5-year survival probabilities in patients with baseline LLV according to user-input clinical variables. The web-based calculators for Model 1 and Model 2 are available online at https://maheng.shinyapps.io/Model1/ and https://maheng.shinyapps.io/Model2/, respectively.

## Discussion

This study has three main findings. First, baseline LLV was associated with poorer OS than baseline HBV DNA <20 IU/mL in patients with HBV-HCC. The association remained evident in the unmatched, PSM, and IPTW analyses. Second, exploratory immune analyses showed a lower peripheral CD4/CD8 ratio in patients with baseline LLV, whereas absolute CD4^+^ and CD8^+^ T cell counts were similar between the two virological groups. Third, because patients with baseline LLV remained clinically heterogeneous, we developed two survival prediction models to refine individualized risk assessment within this population.

Previous studies of LLV have focused predominantly on HCC development rather than prognosis after HCC diagnosis (9–11, 20). Evidence in patients with established HCC remains limited, and existing studies have often been restricted to specific treatment settings or have emphasized longitudinal HBV DNA patterns during follow-up (12–15). Because baseline HBV DNA is routinely available before or at the initiation of HCC treatment, it may provide a practical early marker for risk assessment before longitudinal virological data become available. By focusing on baseline HBV DNA status at initial HCC evaluation, our study therefore extends prior work from HCC risk prediction to post-diagnosis survival in patients with established HCC.

Several mechanisms may explain the association between baseline LLV and poorer survival after HCC diagnosis. Persistent low-level HBV replication can sustain hepatic necroinflammation and hepatocyte injury, potentially accelerating deterioration of hepatic functional reserve (21, 22). In patients with HCC, impaired liver reserve may reduce treatment tolerance, limit eligibility for repeated or intensive antitumor therapies, and compromise post-treatment recovery (17, 23). Detectable HBV DNA at baseline may also indicate incomplete viral suppression and greater susceptibility to virological fluctuation or HBV reactivation during antitumor treatment (24–26). Consistent with this biological framework, our exploratory pathway analyses identified statistically significant indirect effects through ALB and AST, with estimated mediated proportions of 13.4% and 23.1%, respectively, whereas the indirect effects through CHE and ALT were not statistically significant. These findings support the possibility that hepatic functional impairment and hepatocellular injury contribute to the association between residual viral activity and survival. This pathway may be particularly relevant in patients whose prognosis is not overwhelmingly dominated by advanced tumor burden.

Persistent viral activity may also influence systemic inflammation and host immune regulation. In patients with unresectable HBV-HCC receiving immune checkpoint inhibitors, LLV has been associated with poorer treatment response and survival, and proteomic analysis identified differential expression of Flt3L, SLAMF1, and FGF-5 between LLV and maintained virological response groups (13). These observations suggest that residual viral activity may be linked to an immune and inflammatory context relevant to antitumor responses. In our exploratory pathway analysis, SIRI showed a statistically significant indirect effect, with an estimated mediated proportion of 9.6%, providing additional support for a possible role of systemic inflammation. Consistent with this immune context, patients with baseline LLV had a lower peripheral CD4/CD8 ratio than those with HBV DNA <20 IU/mL, whereas absolute CD4^+^ and CD8^+^ T cell counts were similar between the groups. The CD4/CD8 ratio reflects the balance between helper and cytotoxic T cell compartments and may indicate systemic cellular immune competence. CD4^+^ T cell help is important for effective cytotoxic CD8^+^ T cell responses, whereas persistent antigen stimulation can promote CD8^+^ T cell dysfunction and exhaustion (27, 28). Therefore, the lower CD4/CD8 ratio in patients with LLV may reflect an immune imbalance driven by persistent viral activity, which may weaken antitumor immune surveillance, sustain chronic inflammation, and facilitate immune escape. Similar clinical observations have linked peripheral CD4/CD8 imbalance with treatment response or disease progression in other malignancies, including advanced lung cancer and chronic lymphocytic leukemia (29, 30).

The relative importance of viral activity, hepatic reserve, and host immune regulation may vary according to tumor burden and tumor biological activity. This may help explain the subgroup patterns observed in our study. The association was more evident among patients with BCLC stage 0–B disease and AFP ≤400 ng/mL, but less apparent among those with advanced-stage disease or high AFP levels. This pattern may reflect differences in dominant prognostic drivers across disease stages. In patients with lower tumor burden, viral activity and hepatic reserve may remain important contributors to long-term outcomes; in advanced or biologically aggressive disease, tumor progression may dominate prognosis and attenuate the relative contribution of baseline viral status.

Beyond establishing the overall prognostic relevance of baseline LLV, further stratification within this subgroup is clinically important. Prediction model development was motivated by the need to refine individualized risk assessment among patients with baseline LLV. The goal was not only to compare survival between baseline virological groups, but also to characterize prognostic heterogeneity within the baseline LLV population. Two complementary models were therefore developed. Model 1 incorporated tumor-related and laboratory variables and was intended for use when complete clinical and imaging information is available. Model 2 was designed as a simplified non-imaging alternative for settings in which detailed imaging-derived tumor variables are incomplete or difficult to standardize. Thus, Model 1 prioritizes predictive performance, whereas Model 2 emphasizes broader applicability in routine clinical practice.

The retained predictors captured two major prognostic dimensions: tumor-related burden and aggressiveness, as well as hepatic functional reserve and hepatobiliary injury. In Model 1, tumor size, tumor multiplicity, and PVTT reflected structural tumor burden and vascular invasion (31, 32). AFP, retained in both models, represented tumor biological activity beyond anatomic tumor characteristics (33). CHE, ALP, and GGT reflected hepatic synthetic capacity, cholestasis, and hepatobiliary injury (34–36). Together, these variables suggest that survival among patients with baseline LLV is shaped by both tumor-related factors and the underlying hepatic condition.

Consistent with the rationale for dedicated risk stratification, the final RSF implementations of Model 1 and Model 2 showed numerically higher AUCs and generally lower Brier scores than conventional prognostic systems, including Child–Pugh class, BCLC stage, ALBI grade, TNM stage, and MELD score. This finding does not diminish the clinical value of conventional prognostic systems, which remain essential for treatment allocation and broad risk stratification. Instead, the proposed models may provide complementary individualized estimates within the specific subgroup of patients with baseline LLV. To further assess applicability, exploratory analyses across initial HCC treatment groups showed broadly consistent model performance.

Beyond model-based risk prediction, the present study suggests that baseline LLV may serve as a practical marker for identifying patients with HBV-HCC who remain at increased risk. Because patients with baseline LLV had poorer survival, this virological phenotype may prompt closer longitudinal monitoring of HBV DNA and liver function during antitumor therapy, together with reassessment of factors that may contribute to incomplete viral suppression, including adherence, antiviral potency, treatment interruption, and possible virological breakthrough or resistance. For patients with chronic hepatitis B who remain viremic during entecavir therapy, prospective studies have shown that switching to tenofovir alafenamide can improve complete virological and biochemical responses compared with continuing entecavir (37, 38). However, the benefit of switching may not be uniform. In patients with LLV detected only by highly sensitive assays despite negative conventional HBV DNA testing, maintaining or switching nucleos(t)ide analogue therapy resulted in similar virological responses at 48 weeks (39). More recently, the capsid assembly modulator GST-HG141, added to ongoing nucleos(t)ide analogue therapy, produced greater suppression of residual HBV DNA and pregenomic RNA in a phase II trial, although this approach remains investigational (40). Collectively, these studies indicate that the benefit of switching or intensifying antiviral therapy may vary according to the virological and treatment context (37–40).

Importantly, most available rescue studies were conducted in patients with chronic hepatitis B and focused primarily on virological or biochemical endpoints rather than survival after HCC diagnosis (37–40). They therefore do not establish that antiviral switching or treatment intensification improves OS in patients with HBV-HCC. Our findings support recognition of baseline LLV as a marker warranting closer virological assessment, but do not justify routine antiviral switching. Decisions regarding antiviral initiation, optimization, or switching should be guided by existing recommendations and hepatology specialist assessment, particularly in patients with rising HBV DNA, virological breakthrough, or worsening hepatic function (8, 24, 41). Within this framework, the proposed models may further stratify patients with baseline LLV and help identify those who require intensified surveillance and coordinated hepatology and oncology follow-up.

These clinical implications should be interpreted in light of several limitations. First, this was a single-center retrospective study, and residual confounding and selection bias could not be fully excluded. HCC treatment evolved from 2007 to 2017, including changes in systemic and local therapies. These temporal effects could not be fully controlled in this retrospective study and should be considered when interpreting the results. We also evaluated the model’s clinical relevance across treatment groups. The surgical and systemic therapy subgroup analyses were informative but exploratory and limited by small sample sizes. Second, although internal validation was performed, external validation in independent multicenter cohorts is still required before routine clinical application, particularly for the web-based calculators. We consider prospective validation using independent multicenter data an important direction for future research. Third, HBV DNA status was assessed only at baseline, and detailed longitudinal antiviral information, including treatment duration, adherence, antiviral resistance, virological breakthrough, and subsequent treatment modification, was limited. Serial HBV DNA trajectories may provide additional prognostic information and should be incorporated in future prospective studies. Fourth, only the characteristics of peripheral blood T cell subsets and limited immune inflammatory indicators were analyzed, and HBV-specific T cell function or intrahepatic and tumor immune microenvironment were not evaluated. Future studies will further explore the function of immune cells in peripheral blood and tissues of HBV-HCC patients with LLV and possible biological mechanisms.

In conclusion, baseline LLV appears to identify a subgroup with poorer prognosis among patients with HBV-HCC. In these patients, RSF models using routine clinical, tumor, and laboratory variables may support individualized survival prediction. Exploratory analyses suggest that this adverse prognosis may be related to imbalanced T cell subsets and immune and inflammatory dysregulation. Future prospective multicenter studies incorporating longitudinal HBV DNA measurements and immune assessments are needed to validate these findings and explore whether individualized virological management can improve clinical outcomes.

## Data Availability

The raw data supporting the conclusions of this article will be made available by the authors, without undue reservation.
