# Supplementary material for: Baseline low-level viremia in HBV-related hepatocellular carcinoma: prognostic significance, risk stratification, and clinical implications
Source: Front Immunol. 2026 Sep 22;17:1900644. doi: 10.3389/fimmu.2026.1900644 (PMC13640012; doi:10.3389/fimmu.2026.1900644)
Supplement: Supplementary file 1 [file DataSheet1.docx]

**Table**

**Table S1. Study variables, classifications, cutoff values, and roles in the analyses.**

| **Variable** | **Classification / cutoff** | **Role in analysis** |
| --- | --- | --- |
| **Exposure variable** |  |  |
| Baseline HBV DNA status | HBV DNA <20 IU/mL vs baseline LLV, defined as HBV DNA 20–2000 IU/mL | Exposure variable; included in PS model |
| Baseline HBV DNA within the LLV cohort | ≤1600 vs >1600 IU/mL, determined by X-tile | Candidate variable for Model 1 and Model 2 |
| **Demographic characteristics** |  |  |
| Age | ≤60 vs >60 years | PS model; candidate for Model 1 and Model 2 |
| Sex | Male vs female | PS model; candidate for Model 1 and Model 2 |
| **Comorbidities and liver disease background** |  |  |
| Hypertension | No vs yes | PS model; candidate for Model 1 and Model 2 |
| Diabetes mellitus | No vs yes | PS model; candidate for Model 1 and Model 2 |
| Cirrhosis | No vs yes | PS model; candidate for Model 1 and Model 2 |
| **Treatment-related variables** |  |  |
| Antiviral regimen | First-line vs non-first-line antiviral drugs | PS model; candidate for Model 1 and Model 2 |
| Initial HCC treatment | Surgical therapy, minimally invasive therapy, systemic therapy | PS model; candidate for Model 1 |
| **HBV-related virological markers** |  |  |
| Hepatitis B surface antigen (HBsAg) | ≤250 vs >250 IU/mL | PS model; candidate for Model 1 and Model 2 |
| Hepatitis B e antigen (HBeAg) | <1 vs ≥1 IU/mL | PS model; candidate for Model 1 and Model 2 |
| **Tumor burden and staging variables** |  |  |
| Tumor size | <3 cm, 3-5 cm, >5 cm | PS model; candidate for Model 1; final predictor in Model 1 |
| Tumor multiplicity | Solitary vs multiple | PS model; candidate for Model 1; final predictor in Model 1 |
| Portal vein tumor thrombus (PVTT) | No vs yes | PS model; candidate for Model 1; final predictor in Model 1 |
| Barcelona Clinic Liver Cancer (BCLC) stage | 0–B vs C–D | PS model, conventional prognostic system |
| Tumor–node–metastasis (TNM) stage | Ⅰ, Ⅱ, Ⅲ, Ⅳ | conventional prognostic system |
| Alpha-fetoprotein (AFP) | ≤400 vs >400 ng/mL | PS model; candidate for Model 1 and Model 2; final predictor in both models |
| **Hepatic functional reserve and conventional prognostic systems** |  |  |
| Child–Pugh class | A vs B–C | PS model; conventional prognostic system |
| Albumin–bilirubin (ALBI) grade | grade 1, grade 2, grade 3, | Conventional prognostic system |
| Model for End-Stage Liver Disease (MELD) score | <14, 14-18, >18 | Conventional prognostic system |
| **Inflammatory and hematologic indices** |  |  |
| Neutrophil-to-lymphocyte ratio (NLR) | ≤2 vs >2 | PS model; candidate for Model 1 and Model 2; exploratory mediation analysis |
| systemic immune-inflammation response index (SIRI) | <1.05 vs ≥1.05 | Exploratory mediation analysis only |
| Hemoglobin (HGB) | <110 g/L vs ≥110 g/L | PS model; candidate for Model 1 and Model 2 |
| Platelet count (PLT) | ≥100 vs <100 ×10⁹/L | PS model; candidate for Model 1 and Model 2 |
| **Liver injury and cholestasis markers** |  |  |
| Alanine aminotransferase (ALT) | ≤50 vs >50 U/L | PS model; candidate for Model 1 and Model 2; exploratory mediation analysis |
| Aspartate aminotransferase (AST) | ≤40 vs >40 U/L | PS model; candidate for Model 1 and Model 2; exploratory mediation analysis |
| Gamma-glutamyl transferase (GGT) | ≤60 vs >60 U/L | PS model; candidate for Model 1 and Model 2 ; final predictor in Model 2 |
| Alkaline phosphatase (ALP) | ≤125 vs >125 U/L | PS model; candidate for Model 1 and Model 2 ; final predictor in Model 1 and Model 2 |
| Total bilirubin (TBIL) | ≤18.8 vs >18.8 μmol/L | PS model; candidate for Model 1 and Model 2 |
| Albumin (ALB) | ≥40 vs <40 g/L | PS model; candidate for Model 1 and Model 2; exploratory mediation analysis |
| Cholinesterase (CHE) | >4000 vs ≤4000 U/L | PS model; candidate for Model 1 and Model 2 ; final predictor in Model 1 and Model 2; exploratory mediation analysis |
| **Renal function, coagulation, and tissue injury markers** |  |  |
| Serum creatinine (Cr) | ≤111 vs >111 μmol/L | PS model; candidate for Model 1 and Model 2 |
| International normalized ratio (INR) | ≤1.5 vs >1.5 | PS model; candidate for Model 1 and Model 2 |
| Lactate dehydrogenase (LDH) | ≤250 vs >250 U/L | PS model; candidate for Model 1 and Model 2 |
| Peripheral cellular immune parameters |  |  |
| Absolute CD4⁺ T-cell count | Continuous | Exploratory peripheral T-cell analysis only |
| Absolute CD8⁺ T-cell count | Continuous | Exploratory peripheral T-cell analysis only |
| CD4/CD8 ratio | Continuous | Exploratory peripheral T-cell analysis only |

Note: Abbreviations are defined at first mention in the table. Initial HCC treatment refers to the first HCC-directed treatment modality at baseline.

**Table S2. Hyperparameter search spaces and selected values for the machine-learning algorithms used in Model 1 and Model 2.**

| **Algorithm** | **Hyperparameter** | **Search space** | **Model 1**  **(6 predictors)** | **Model 2**  **(4 predictors)** |
| --- | --- | --- | --- | --- |
| **SVM** | gamma.mu | 0.02, 0.03, 0.04, 0.05, 0.10, 0.25, 0.50 | 0.02 | 0.02 |
|  | diff.meth | makediff1, makediff3 | makediff1 | makediff1 |
| **GBM** | n.trees | 300, 500, 800, 1000, 1500, 2000 | 1500 | 500 |
|  | interaction.depth | 1, 2 | 2 | 2 |
|  | shrinkage | 0.003, 0.005, 0.010, 0.020 | 0.005 | 0.010 |
|  | n.minobsinnode | 15, 20, 30, 40 | 20 | 20 |
| **RSF** | ntree | 500, 1000, 1500, 2000, 3000 | 500 | 500 |
|  | mtry | Model 1: 2-5; Model 2: 2-4 | 2 | 2 |
|  | nodesize | 10, 15, 20, 25, 30, 40 | 30 | 30 |
| **XGBoost** | max_depth | 1, 2, 3 | 2 | 1 |
|  | eta | 0.005, 0.010, 0.020, 0.030 | 0.020 | 0.020 |
|  | nrounds | 300, 600, 1000, 1500, 2000 | 173* | 346* |

**Note:** Model 1 was based on six selected predictors, whereas Model 2 was based on four selected predictors. Hyperparameters were optimized within the training set. Candidate values shown represent the tuning grids. For RSF, mtry ranged from 2 to 5 for Model 1 and from 2 to 4 for Model 2 according to the number of available predictors. For XGBoost, the final number of boosting iterations was determined by early stopping during training. SVM, GBM, RSF, and XGBoost were implemented using the survivalsvm (version 0.0.5), gbm (version 2.2.2), randomForestSRC (version 3.3.2), and xgboost (version 1.7.8.1) R packages, respectively. Analyses were performed in R version 4.0.5. A fixed random seed of 123 was used throughout model development to ensure reproducibility.

**Abbreviations:** GBM, gradient boosting machine; RSF, random survival forest; SVM, support vector machine; XGBoost, extreme gradient boosting.

**Table S3. Baseline characteristics according to baseline HBV DNA status after inverse probability of treatment weighting.**

| **Variable** | **IPTW Cohort** | | | |
| --- | --- | --- | --- | --- |
|  | **20-2000IU/mL ( Weighted )** | **<20IU/mL ( Weighted )** | **P** | **SMD** |
| n | 250.7 | 310.0 |  |  |
| **Sex = male (%)** | 194.3(77.5) | 241.0 (77.7) | 0.947 | 0.005 |
| **Age >60 (%)** | 86.0 (34.3) | 113.0(36.5) | 0.577 | 0.044 |
| **Hypertension = yes (%)** | 63.9 (25.5) | 77.0 (24.8) | 0.850 | 0.015 |
| **Diabetes = yes (%)** | 56.8 (22.7) | 77.0 (24.8) | 0.525 | 0.051 |
| **Cirrhosis = yes (%)** | 240.1(95.8) | 300.0(96.8) | 0.472 | 0.052 |
| **BCLC = C-D (%)** | 44.9 (17.9) | 48.0 (15.5) | 0.378 | 0.065 |
| **Child-Pugh = B-C (%)** | 72.8 (29.0) | 85.0 (27.4) | 0.649 | 0.036 |
| **HBsAg >250IU/mL (%)** | 157.7(62.9) | 172.0(55.5) | 0.062 | **0.152** |
| **HBeAg ≥1IU/mL (%)** | 65.8 (26.2) | 74.0 (23.9) | 0.478 | 0.055 |
| **Antiviral regimen = First-line drugs (%)** | 167.1(66.7) | 208.0(67.1) | 0.910 | 0.009 |
| **Initial HCC treatment (%)** |  |  | 0.911 | 0.034 |
| Systemic Therapy | 22.7 (9.0) | 31.0 (10.0) |  |  |
| Minimally Invasive Therapy | 203.0(81.0) | 249.0(80.3) |  |  |
| Surgical Therapy | 25.1 (10.0) | 30.0 (9.7) |  |  |
| **Tumor size (%)** |  |  | 0.183 | **0.142** |
| <3cm | 131.5(52.5) | 184.0(59.4) |  |  |
| 3-5cm | 70.6 (28.2) | 77.0 (24.8) |  |  |
| >5cm | 48.6 (19.4) | 49.0 (15.8) |  |  |
| **Tumor multiplicity = Multiple (%)** | 104.6(41.7) | 115.0(37.1) | 0.228 | 0.095 |
| **PVTT = yes (%)** | 31.2 (12.4) | 35.0 (11.3) | 0.623 | 0.035 |
| **NLR >2 (%)** | 145.5(58.0) | 170.0 (54.8) | 0.418 | 0.064 |
| **HGB <110g/L (%)** | 59.3 (23.7) | 79.0 (25.5) | 0.598 | 0.042 |
| **PLT <100 × 10^9/L (%)** | 144.7(57.7) | 181.0 (58.4) | 0.862 | 0.014 |
| **Cr >111μmol/L (%)** | 8.7 (3.5) | 14.0 (4.5) | 0.514 | 0.053 |
| **ALT >50U/L (%)** | 33.7(13.4) | 34.0(11.0) | 0.298 | 0.076 |
| **AST >40U/L (%)** | 71.1(28.4) | 70.0(22.6) | 0.073 | **0.133** |
| **TBIL >18.8umol/L (%)** | 104.5(41.7) | 117.0(37.7) | 0.306 | 0.081 |
| **ALB <40g/L (%)** | 158.5(63.2) | 185.0(59.7) | 0.363 | 0.073 |
| **LDH >250U/L (%)** | 19.9 (7.9) | 27.0 (8.7) | 0.702 | 0.028 |
| **GGT >60U/L (%)** | 86.5 (34.5) | 96.0 (31.0) | 0.326 | 0.075 |
| **ALP >125U/L (%)** | 49.3 (19.7) | 54.0 (17.4) | 0.447 | 0.058 |
| **CHE >4000U/L (%)** | 173.2(69.1) | 219.0(70.6) | 0.662 | 0.034 |
| **INR >1.5 (%)** | 13.0 (5.2) | 17.0 (5.5) | 0.871 | 0.013 |
| **AFP >400ng/mL (%)** | 33.1 (13.2) | 33.0 (10.6) | 0.275 | 0.079 |

Note: Values are presented as weighted No. (%) unless otherwise indicated. HBV DNA groups are expressed in IU/mL; 20–2000 IU/mL represents baseline low-level viremia. Covariate balance after inverse probability of treatment weighting was assessed using standardized mean differences, with values <0.1 indicating acceptable balance. Initial HCC treatment refers to the first HCC-directed treatment modality at baseline.

Abbreviations: BCLC, Barcelona Clinic Liver Cancer; HBsAg, hepatitis B surface antigen; HBeAg, hepatitis B e antigen; HCC, hepatocellular carcinoma; PVTT, portal vein tumor thrombus; NLR, neutrophil-to-lymphocyte ratio; HGB, hemoglobin; PLT, platelet count; Cr, creatinine; ALT, alanine aminotransferase; AST, aspartate aminotransferase; TBIL, total bilirubin; ALB, albumin; LDH, lactate dehydrogenase; GGT, gamma-glutamyl transferase; ALP, alkaline phosphatase; CHE, cholinesterase; INR, international normalized ratio; AFP, alpha-fetoprotein; IPTW, inverse probability of treatment weighting; SMD, standardized mean difference.

**Table S4. Multicollinearity diagnostics for covariates in the primary multivariable Cox model.**

| **Variable** | **VIF** | **Tolerance** |
| --- | --- | --- |
| **HBV DNA status** | 1.330 | 0.752 |
| **Sex** | 1.139 | 0.878 |
| **Age** | 1.223 | 0.817 |
| **Hypertension** | 1.202 | 0.832 |
| **Diabetes** | 1.187 | 0.843 |
| **Cirrhosis** | 1.094 | 0.914 |
| **BCLC** | 2.694 | 0.371 |
| **Child–Pugh** | 1.845 | 0.542 |
| **HBsAg** | 1.099 | 0.910 |
| **HBeAg** | 1.133 | 0.883 |
| **Antiviral regimen** | 1.140 | 0.877 |
| **Initial HCC treatment** | 1.344 | 0.744 |
| **Tumor size** | 1.419 | 0.705 |
| **Tumor multiplicity** | 1.146 | 0.873 |
| **PVTT** | 2.406 | 0.416 |
| **NLR** | 1.161 | 0.861 |
| **HGB** | 1.497 | 0.668 |
| **PLT** | 1.343 | 0.745 |
| **Cr** | 1.144 | 0.874 |
| **ALT** | 1.327 | 0.754 |
| **AST** | 1.674 | 0.597 |
| **TBIL** | 1.640 | 0.610 |
| **ALB** | 1.477 | 0.677 |
| **LDH** | 1.668 | 0.599 |
| **GGT** | 1.537 | 0.651 |
| **ALP** | 1.162 | 0.861 |
| **CHE** | 1.756 | 0.569 |
| **INR** | 1.310 | 0.763 |
| **AFP** | 1.219 | 0.821 |

**Note:** Multicollinearity among covariates included in the primary multivariable Cox proportional hazards model was assessed using variance inflation factors (VIFs) and tolerance. No substantial multicollinearity was identified, with all VIF values <3 and all tolerance values >0.3.

**Table S5. Proportional hazards diagnostics and corresponding sensitivity-analysis strategies for multivariable Cox models.**

**Panel A. Initial proportional hazards diagnostics**

| **Analysis** | **HBV DNA PH P** | **Global PH P** | **Covariates with PH violation (P<0.05)** |
| --- | --- | --- | --- |
| Unmatched multivariable Cox | 0.119 | <0.001 | BCLC; Child-Pugh; initial HCC treatment; tumor size; PVTT; NLR; PLT; ALT; AST; TBIL; GGT; ALP; LDH; CHE; AFP |
| PSM multivariable Cox | 0.051 | 0.027 | Child-Pugh; tumor size; PLT; LDH; ALP |
| IPTW multivariable Cox | 0.825 | 0.003 | Child-Pugh; initial HCC treatment; tumor size; NLR; PLT; AST; LDH; GGT; ALP; AFP |
| IPTW model additionally adjusted for residual imbalance | 0.661 | <0.001 | Tumor size; AST |

**Panel B. Proportional hazards diagnostics after stratified sensitivity analysis**

| **Analysis** | **Stratification factors** | **HBV DNA PH P after stratification** | **Global PH P after stratification** | **Residual PH violations** |
| --- | --- | --- | --- | --- |
| Unmatched multivariable Cox | BCLC; Child-Pugh; initial HCC treatment; tumor size; PVTT; AST; GGT; ALP; AFP | 0.078 | 0.066 | Age (P=0.040); PLT (P=0.002) |
| PSM multivariable Cox | Child-Pugh; tumor size; PLT; LDH; ALP | 0.222 | 0.639 | None |
| IPTW multivariable Cox | Child-Pugh; tumor size; PLT; LDH; ALP; AFP | 0.480 | 0.720 | None |
| IPTW model additionally adjusted for residual imbalance | Tumor size; AST | 0.360 | 0.610 | None |

**Panel C.** **Association estimates after stratified sensitivity analysis**

| **Analysis** | **HR** | **95% CI** | **P value** |
| --- | --- | --- | --- |
| Unmatched multivariable Cox | 0.749 | 0.580-0.966 | 0.026 |
| PSM multivariable Cox | 0.577 | 0.451–0.739 | <0.001 |
| IPTW multivariable Cox | 0.610 | 0.492–0.757 | <0.001 |
| IPTW model additionally adjusted for residual imbalance | 0.713 | 0.587–0.867 | <0.001 |

**Note:** The proportional hazards (PH) assumption was assessed using Schoenfeld residuals. A P value <0.05 was considered evidence of potential non-proportional hazards. Panel A summarizes the initial PH diagnostics. Panel B summarizes the stratified sensitivity analyses, in which selected covariates showing evidence of non-proportional hazards were incorporated as stratification factors to allow stratum-specific baseline hazards. The PH assumption was reassessed after stratification. Baseline HBV DNA status did not show evidence of PH violation in any of the original models. In the unmatched sensitivity model, the global PH test was no longer significant, although residual evidence of non-proportionality remained for age and platelet count.

**Abbreviations:** AFP, alpha-fetoprotein; ALP, alkaline phosphatase; ALT, alanine aminotransferase; AST, aspartate aminotransferase; BCLC, Barcelona Clinic Liver Cancer; CHE, cholinesterase; GGT, gamma-glutamyl transferase; HCC, hepatocellular carcinoma; HBV, hepatitis B virus; IPTW, inverse probability of treatment weighting; LDH, lactate dehydrogenase; NLR, neutrophil-to-lymphocyte ratio; PH, proportional hazards; PLT, platelet count; PSM, propensity score matching; PVTT, portal vein tumor thrombus; TBIL, total bilirubin.

**Table S6. Sensitivity analyses excluding initial HCC treatment from the propensity-score and multivariable adjustment sets.**

**Panel A. Association between baseline HBV DNA status and overall survival after excluding initial HCC treatment**

| **Analysis** | **HR** | **95% CI** | **P value** |
| --- | --- | --- | --- |
| Unmatched multivariable Cox | 0.761 | 0.623-0.930 | 0.007 |
| PSM univariable Cox | 0.771 | 0.617-0.963 | 0.022 |
| PSM multivariable Cox | 0.703 | 0.551-0.898 | 0.005 |
| IPTW univariable Cox | 0.693 | 0.567-0.847 | <0.001 |
| IPTW multivariable Cox | 0.722 | 0.586-0.889 | 0.001 |

**Panel B. Covariate balance after re-estimation of propensity scores excluding initial HCC treatment**

| **Covariate** | **Post-PSM absolute SMD** | **Post-IPTW absolute SMD** |
| --- | --- | --- |
| Sex | 0.039 | 0.004 |
| Age | 0.053 | 0.043 |
| Hypertension | 0.067 | 0.029 |
| Diabetes | 0.080 | 0.034 |
| Cirrhosis | 0.097 | 0.028 |
| BCLC | 0.033 | 0.025 |
| Child-Pugh class | 0.056 | 0.018 |
| HBsAg | <0.001 | 0.097 |
| HBeAg | 0.037 | 0.027 |
| Antiviral regimen | 0.035 | 0.005 |
| Tumor size | 0.050 | 0.091 |
| Tumor multiplicity | 0.034 | 0.065 |
| PVTT | 0.065 | 0.001 |
| NLR | 0.017 | 0.057 |
| HGB | 0.039 | 0.034 |
| PLT | 0.050 | 0.002 |
| Cr | 0.049 | 0.050 |
| ALT | <0.001 | 0.038 |
| AST | <0.001 | 0.068 |
| TBIL | 0.017 | 0.063 |
| ALB | 0.035 | 0.060 |
| LDH | 0.078 | 0.043 |
| GGT | 0.088 | 0.042 |
| ALP | 0.054 | 0.037 |
| CHE | 0.036 | 0.020 |
| INR | <0.001 | 0.011 |
| AFP | 0.012 | 0.042 |

**Sensitivity PSM matched sample:** 241 pairs. Maximum post-adjustment absolute SMD: 0.097 after PSM and 0.097 after IPTW; no modeled covariate had an absolute SMD >0.10 after either adjustment.

Note: In this sensitivity analysis, initial HCC treatment was excluded from the propensity-score and multivariable adjustment sets; propensity scores were re-estimated and multivariable Cox models were refitted accordingly. Absolute standardized mean differences <0.10 were considered indicative of acceptable covariate balance. Initial HCC treatment is not included in Panel B because it was intentionally excluded from the sensitivity propensity-score model and may represent a post-exposure variable.

**Abbreviations:** AFP, alpha-fetoprotein; ALB, albumin; ALP, alkaline phosphatase; ALT, alanine aminotransferase; AST, aspartate aminotransferase; BCLC, Barcelona Clinic Liver Cancer; CHE, cholinesterase; CI, confidence interval; Cr, creatinine; GGT, gamma-glutamyl transferase; HBeAg, hepatitis B e antigen; HBsAg, hepatitis B surface antigen; HCC, hepatocellular carcinoma; HGB, hemoglobin; HR, hazard ratio; INR, international normalized ratio; IPTW, inverse probability of treatment weighting; LDH, lactate dehydrogenase; NLR, neutrophil-to-lymphocyte ratio; PLT, platelet count; PSM, propensity score matching; PVTT, portal vein tumor thrombus; SMD, standardized mean difference; TBIL, total bilirubin.

**Table S7. Availability and exploratory analysis of peripheral T-cell phenotyping according to baseline HBV DNA status.**

| **Immune parameter** | **LLV**  **(n=362)** | **HBV DNA <20 IU/mL**  **(n=169)** | **P value** | **FDR-adjusted**  **P value** |
| --- | --- | --- | --- | --- |
| Absolute CD4+ T-cell count | 475.50 (302.75-672.50) | 462.00 (303.00-693.00) | 0.684 | 0.684 |
| Absolute CD8+ T-cell count | 279.00 (162.00-432.25) | 256.00 (156.00-365.00) | 0.185 | 0.278 |
| CD4/CD8 ratio | 1.69 (1.29-2.22) | 1.87 (1.36-2.67) | 0.016 | 0.047 |

**Note:** Values are presented as median (interquartile range) and were compared using the Mann–Whitney U test. P values were adjusted for multiple comparisons using the Benjamini–Hochberg false discovery rate (FDR) procedure.

**Abbreviations:** FDR, false discovery rate; HBV, hepatitis B virus; LLV, low-level viremia.

**Table S8. Baseline Characteristics of the Training and Validation Sets.**

| **Variable** | **Training Set** | **Validation Set** | **P Value** |
| --- | --- | --- | --- |
| **Event** | 345 (80.6%) | 139 (76.0%) | 0.194 |
| **Overall survival, months** |  |  | 0.900 |
| **Mean ± SD** | 39.1 ± 38.4 | 38.7 ± 39.9 |  |
| **Median (Q1, Q3)** | 25.0 (6.1, 67.3) | 22.3 (7.3, 63.5) |  |
| **Sex = male (%)** | 338 (79.0%) | 131 (71.6%) | 0.059 |
| **Age >60 (%)** | 128 (29.9%) | 62 (33.9%) | 0.341 |
| **Hypertension = yes (%)** | 98 (22.9%) | 41 (22.4%) | 0.917 |
| **Diabetes = yes (%)** | 80 (18.7%) | 32 (17.5%) | 0.820 |
| **Cirrhosis = yes (%)** | 395 (92.3%) | 167 (91.3%) | 0.745 |
| **BCLC = C-D (%)** | 158 (36.9%) | 59 (32.2%) | 0.310 |
| **Child-Pugh = B-C (%)** | 144 (33.6%) | 67 (36.6%) | 0.516 |
| **HBV DNA >1600 IU/mL (%)** | 41 (9.6%) | 13 (7.1%) | 0.355 |
| **HBsAg >250IU/mL (%)** | 321 (75.0%) | 132 (72.1%) | 0.481 |
| **HBeAg ≥1IU/mL (%)** | 151 (35.3%) | 63 (34.4%) | 0.854 |
| **Antiviral regimen = First-line drugs (%)** | 272 (63.6%) | 126 (68.9%) | 0.229 |
| **Initial HCC treatment (%)** |  |  | 0.814 |
| Systemic Therapy | 57 (13.3%) | 21 (11.5%) |  |
| Minimally Invasive Therapy | 336 (78.5%) | 148 (80.9%) |  |
| Surgical Therapy | 35 (8.2%) | 14 (7.7%) |  |
| **Tumor size (%)** |  |  | 0.802 |
| <3cm | 143 (33.4%) | 56 (30.6%) |  |
| 3-5cm | 123 (28.7%) | 55 (30.1%) |  |
| >5cm | 162 (37.9%) | 72 (39.3%) |  |
| **Tumor multiplicity = Multiple (%)** | 229 (53.5%) | 100 (54.6%) | 0.859 |
| **PVTT = yes (%)** | 101 (23.6%) | 36 (19.7%) | 0.341 |
| **NLR >2 (%)** | 278 (65.0%) | 115 (62.8%) | 0.645 |
| **HGB <110g/L (%)** | 101 (23.6%) | 44 (24.0%) | 0.918 |
| **PLT <100 × 10^9/L (%)** | 217 (50.7%) | 99 (54.1%) | 0.480 |
| **Cr >111μmol/L (%)** | 12 (2.8%) | 4 (2.2%) | 0.787 |
| **ALT >50U/L (%)** | 108 (25.2%) | 49 (26.8%) | 0.687 |
| **AST >40U/L (%)** | 240 (56.1%) | 105 (57.4%) | 0.790 |
| **TBIL >18.8umol/L (%)** | 215 (50.2%) | 83 (45.4%) | 0.290 |
| **ALB <40g/L (%)** | 302 (70.6%) | 125 (68.3%) | 0.630 |
| **LDH >250U/L (%)** | 48 (11.2%) | 21 (11.5%) | >0.999 |
| **GGT >60U/L (%)** | 232 (54.2%) | 101 (55.2%) | 0.859 |
| **ALP >125U/L (%)** | 135 (31.5%) | 54 (29.5%) | 0.634 |
| **CHE >4000U/L (%)** | 253 (59.1%) | 111 (60.7%) | 0.787 |
| **INR >1.5 (%)** | 29 (6.8%) | 13 (7.1%) | 0.863 |
| **AFP >400ng/mL (%)** | 122 (28.5%) | 47 (25.7%) | 0.491 |

**Note:** Values are presented as No. (%) unless otherwise indicated. The training and validation sets were randomly generated from patients with baseline low-level viremia at a 7:3 ratio. Initial HCC treatment refers to the first HCC-directed treatment modality at baseline. Because missing data were imputed separately for association analyses and prediction modeling, counts for variables with missing values may differ slightly between Table 1 and Table S8.

**Abbreviations:** BCLC, Barcelona Clinic Liver Cancer; HBsAg, hepatitis B surface antigen; HBeAg, hepatitis B e antigen; HCC, hepatocellular carcinoma; PVTT, portal vein tumor thrombus; NLR, neutrophil-to-lymphocyte ratio; HGB, hemoglobin; PLT, platelet count; Cr, creatinine; ALT, alanine aminotransferase; AST, aspartate aminotransferase; TBIL, total bilirubin; ALB, albumin; LDH, lactate dehydrogenase; GGT, gamma-glutamyl transferase; ALP, alkaline phosphatase; CHE, cholinesterase; INR, international normalized ratio; AFP, alpha-fetoprotein.

**Table S9. Training set and Validation set Brier score comparison of survival models using 1,000 bootstrap replicates.**

| **Model** | **Parameters** | **Training set** | | | **Validation set** | | |
| --- | --- | --- | --- | --- | --- | --- | --- |
|  |  | **12-month** | **36-month** | **60-month** | **12-month** | **36-month** | **60-month** |
| **Cox** | 6 | 0.171(0.151-0.192) | 0.185(0.167-0.202) | 0.171(0.152-0.190) | 0.167(0.157-0.179) | 0.202(0.187-0.223) | 0.188(0.175-0.205) |
| **GBM** | 6 | 0.158(0.135-0.182) | 0.171(0.145-0.185) | 0.156(0.133-0.180) | 0.160(0.130-0.189) | 0.187(0.158-0.215) | 0.179(0.150-0.211) |
| **SVM** | 6 | 0.179(0.164-0.195) | 0.194(0.178-0.211) | 0.172(0.155-0.192) | 0.175(0.147-0.204) | 0.193(0.167-0.219) | 0.182(0.155-0.210) |
| **RSF** | 6 | 0.157(0.140-0.173) | 0.167(0.149-0.185) | 0.151(0.132-0.169) | 0.161(0.154-0.169) | 0.190(0.184-0.196) | 0.179(0.173-0.186) |
| **XGB** | 6 | 0.169(0.148-0.187) | 0.188(0.171-0.207) | 0.173(0.155-0.204) | 0.164(0.152-0.179) | 0.204(0.187-0.240) | 0.191(0.174-0.234) |
| **Cox** | 4 | 0.183(0.164-0.202) | 0.199(0.182-0.216) | 0.179(0.160-0.197) | 0.187(0.181-0.194) | 0.201(0.196-0.208) | 0.184(0.180-0.190) |
| **GBM** | 4 | 0.179(0.155-0.201) | 0.196(0.173-0.218) | 0.177(0.152-0.202) | 0.188(0.163-0.216) | 0.203(0.177-0.231) | 0.185(0.158-0.214) |
| **SVM** | 4 | 0.188(0.174-0.203) | 0.209(0.194-0.224) | 0.187(0.170-0.207) | 0.179(0.153-0.206) | 0.199(0.174-0.225) | 0.184(0.158-0.211) |
| **RSF** | 4 | 0.174(0.156-0.193) | 0.193(0.175-0.210) | 0.174(0.156-0.193) | 0.179(0.174-0.186) | 0.198(0.191-0.209) | 0.184(0.179-0.192) |
| **XGB** | 4 | 0.194(0.170-0.223) | 0.221(0.195-0.258) | 0.215(0.179-0.269) | 0.206(0.186-0.238) | 0.241(0.212-0.296) | 0.222(0.191-0.287) |

**Note:** Lower Brier score indicates better calibration. Values are mean (95% confidence interval).

**Abbreviations:** GBM = Gradient Boosting Machine; RSF = Random Survival Forest; SVM = Support Vector Machine; XGBoost = XGBoost; Cox = Cox Proportional Hazards

**Table S10. Comparison of time-dependent AUCs and Brier scores for the final RSF-based prediction models and conventional prognostic systems in the training and validation sets.**

| **Model** | **Training set** | | | **Validation set** | | |
| --- | --- | --- | --- | --- | --- | --- |
|  | **12-month** | **36-month** | **60-month** | **12-month** | **36-month** | **60-month** |
| **Panel A. Time-dependent AUC** | | | | | | |
| **Model 1** | 0.842(0.804-0.877) | 0.825(0.782-0.862) | 0.805(0.761-0.846) | 0.824(0.800-0.839) | 0.777(0.763-0.790) | 0.765(0.750-0.778) |
| **Model 2** | 0.785(0.739-0.826) | 0.755(0.710-0.802) | 0.729(0.675-0.777) | 0.769(0.741-0.788) | 0.742(0.709-0.757) | 0.723(0.694-0.735) |
| **BCLC** | 0.750(0.706-0.795) | 0.736(0.691-0.780) | 0.712(0.660-0.759) | 0.751(0.679-0.823) | 0.721(0.653-0.790) | 0.712(0.642-0.781) |
| **TNM** | 0.707(0.659-0.756) | 0.708(0.656-0.752) | 0.700(0.645-0.749) | 0.741(0.664-0.812) | 0.732(0.665-0.799) | 0.713(0.642-0.782) |
| **Child-Pugh** | 0.638(0.592-0.685) | 0.602(0.560-0.646) | 0.599(0.557-0.643) | 0.663(0.589-0.732) | 0.671(0.606-0.730) | 0.641(0.576-0.702) |
| **MELD** | 0.538(0.489-0.575) | 0.517(0.490-0.546) | 0.529(0.497-0.559) | 0.530(0.474-0.581) | 0.536(0.465-0.582) | 0.533(0.466-0.575) |
| **ALBI** | 0.630(0.583-0.677) | 0.608(0.560-0.655) | 0.595(0.542-0.648) | 0.573(0.500-0.649) | 0.659(0.583-0.728) | 0.651(0.567-0.726) |
| **Panel B. Time-dependent Brier score** | | | | | | |
| **Model 1** | 0.157(0.140-0.173) | 0.167(0.149-0.185) | 0.151(0.132-0.169) | 0.161(0.154-0.169) | 0.190(0.184-0.196) | 0.179(0.173-0.186) |
| **Model 2** | 0.174(0.156-0.193) | 0.193(0.175-0.210) | 0.174(0.156-0.193) | 0.179(0.174-0.186) | 0.198(0.191-0.209) | 0.184(0.179-0.192) |
| **BCLC** | 0.241(0.221-0.262) | 0.279(0.263-0.297) | 0.268(0.241-0.294) | 0.242(0.228-0.258) | 0.305(0.271-0.340) | 0.271(0.233-0.312) |
| **TNM** | 0.251(0.231-0.273) | 0.288(0.269-0.309) | 0.271(0.241-0.303) | 0.249(0.233-0.266) | 0.311(0.273-0.354) | 0.277(0.236-0.324) |
| **Child-Pugh** | 0.218(0.202-0.231) | 0.235(0.224-0.244) | 0.202(0.183-0.219) | 0.217(0.212-0.222) | 0.224(0.216-0.236) | 0.199(0.195-0.206) |
| **MELD** | 0.235(0.220-0.250) | 0.251(0.236-0.273) | 0.214(0.189-0.245) | 0.235(0.230-0.248) | 0.251(0.234-0.288) | 0.218(0.207-0.249) |
| **ALBI** | 0.229(0.215-0.242) | 0.245(0.235-0.255) | 0.214(0.193-0.234) | 0.234(0.228-0.243) | 0.244(0.228-0.266) | 0.212(0.200-0.232) |

**Note:** Panel A presents time-dependent AUCs, and Panel B presents time-dependent Brier scores. Model 1 and Model 2 represent the final random survival forest implementations developed from the full candidate-variable set and the non-imaging candidate-variable set, respectively. AUCs and Brier scores were evaluated at 12, 36, and 60 months. Values are presented as bootstrap means (95% confidence intervals) based on 1,000 resamples. Higher AUC values indicate better discrimination, whereas lower Brier scores indicate lower prediction error.

**Abbreviations:** AFP, alpha-fetoprotein; ALBI, albumin–bilirubin; ALP, alkaline phosphatase; AUC, area under the curve; BCLC, Barcelona Clinic Liver Cancer; CHE, cholinesterase; GGT, gamma-glutamyl transferase; MELD, Model for End-Stage Liver Disease; PVTT, portal vein tumor thrombus; RSF, random survival forest; TNM, tumor–node–metastasis.

**Table S11. Exploratory assessment of time-dependent AUCs and Brier scores for Model 1 and Model 2 in the overall baseline LLV cohort and initial HCC treatment subgroups.**

| **Population** | **Sample size (n)** | **Model 1** | | | **Model 2** | | |
| --- | --- | --- | --- | --- | --- | --- | --- |
|  |  | **12-month** | **36-month** | **60-month** | **12-month** | **36-month** | **60-month** |
| **Panel A. Time-dependent AUC** | | | | | | | |
| **Surgical therapy** | 49 | 0.871(0.845-0.891) | 0.766(0.730-0.797) | 0.678(0.635-0.714) | 0.813(0.787-0.833) | 0.778(0.761-0.786) | 0.723(0.701-0.736) |
| **Systemic therapy** | 78 | 0.735(0.701-0.758) | 0.804(0.770-0.835) | 0.768(0.731-0.808) | 0.720(0.692-0.745) | 0.796(0.767-0.820) | 0.774(0.740-0.794) |
| **Minimally Invasive therapy** | 484 | 0.815(0.798-0.825) | 0.781(0.771-0.788) | 0.776(0.762-0.787) | 0.748(0.722-0.762) | 0.710(0.696-0.717) | 0.693(0.676-0.700) |
| **Overall** | 611 | 0.827(0.814-0.836) | 0.799(0.792-0.805) | 0.782(0.773-0.792) | 0.773(0.754-0.783) | 0.743(0.729-0.749) | 0.721(0.705-0.727) |
| **Panel B. Time-dependent Brier score** | | | | | | | |
| **Surgical therapy** | 49 | 0.121(0.113-0.130) | 0.209(0.191-0.229) | 0.255(0.235-0.277) | 0.138(0.128-0.152) | 0.207(0.189-0.227) | 0.250(0.230-0.272) |
| **Systemic therapy** | 78 | 0.206(0.190-0.225) | 0.114(0.104-0.126) | 0.091(0.084-0.099) | 0.214(0.197-0.234) | 0.118(0.107-0.133) | 0.090(0.084-0.099) |
| **Minimally Invasive therapy** | 484 | 0.161(0.157-0.166) | 0.187(0.184-0.192) | 0.168(0.165-0.172) | 0.179(0.176-0.185) | 0.212(0.209-0.216) | 0.189(0.187-0.193) |
| **Overall** | 611 | 0.163(0.160-0.169) | 0.180(0.177-0.184) | 0.165(0.162-0.168) | 0.181(0.177-0.185) | 0.199(0.197-0.204) | 0.181(0.179-0.185) |

**Note:** Panel A presents time-dependent AUCs, and Panel B presents time-dependent Brier scores. Model 1 and Model 2 represent the final random survival forest implementations developed from the full candidate-variable set and the non-imaging candidate-variable set, respectively. Model performance was evaluated at 12, 36, and 60 months. Values are presented as bootstrap means (95% confidence intervals) based on 1,000 resamples. Higher AUC values indicate better discriminative performance, whereas lower Brier scores indicate lower prediction error. Analyses across initial HCC treatment groups were exploratory and were not intended as independent validation of the models.

**Abbreviations:** AUC, area under the curve; HCC, hepatocellular carcinoma; LLV, low-level viremia; RSF, random survival forest.

**Figure:**

Figure S1. Missing rates of baseline variables according to baseline HBV DNA status. (A) Patients with baseline low-level viremia. (B) Patients with baseline HBV DNA <20 IU/mL.

**Figure S2. Propensity score distributions before and after propensity score matching.** Panels A and C show the propensity score distributions before matching for patients with baseline HBV DNA <20 IU/mL and those with baseline low-level viremia (LLV), respectively. Panels B and D show the corresponding distributions after 1:1 nearest-neighbor propensity score matching without replacement. Propensity scores were estimated using the prespecified covariates included in the propensity score model. Vertical dashed lines indicate the boundaries of the common support region (approximately 0.040–0.872). Histograms are presented as proportions within each group.

**Figure S3. Exploratory mediation analyses of potential pathways linking baseline low-level viremia to overall survival.** Panels A–F show mediation analyses for cholinesterase (CHE), albumin (ALB), aspartate aminotransferase (AST), alanine aminotransferase (ALT), neutrophil-to-lymphocyte ratio (NLR), and systemic immune-inflammation response index (SIRI), respectively. Candidate mediators were defined as CHE ≤4000 U/L, ALB <40 g/L, AST >40 U/L, ALT >50 U/L, NLR >2, and SIRI ≥1.05. Baseline LLV was treated as the exposure, with HBV DNA <20 IU/mL as the reference. ORs describe the associations between baseline LLV and the candidate mediators, whereas HRs describe associations with mortality and the estimated natural indirect, natural direct, and total effects. Models were adjusted for age, sex, cirrhosis, diabetes, hypertension, antiviral regimen, tumor size, tumor multiplicity, HBeAg, HBsAg, PVTT, and AFP. Values in parentheses indicate 95% confidence intervals. The proportion mediated represents the estimated percentage of the total association statistically accounted for by each mediator.

**Figure S4. Selection of prognostic features for Model 2 from non-imaging candidate variables.** (A) Least absolute shrinkage and selection operator Cox regression coefficient profiles. (B) Ten-fold cross-validation for selecting the optimal lambda in the LASSO-Cox model. (C) Variable importance ranking generated by the Boruta algorithm.
